# Identification and characterization of SSE15206, a microtubule depolymerizing agent that overcomes multidrug resistance

**DOI:** 10.1038/s41598-018-21642-0

**Published:** 2018-02-19

**Authors:** Safia Manzoor, Aishah Bilal, Sardraz Khan, Rahim Ullah, Sunniya Iftikhar, Abdul-Hamid Emwas, Meshari Alazmi, Xin Gao, Ali Jawaid, Rahman Shah Zaib Saleem, Amir Faisal

**Affiliations:** 1 Department of Chemistry, Syed Babar Ali School of Science and Engineering, Lahore University of Management Sciences, Lahore, 54792 Pakistan; 2 Department of Biology, Syed Babar Ali School of Science and Engineering, Lahore University of Management Sciences, Lahore, 54792 Pakistan; 3 Imaging and Characterization Core Lab, King Abdullah University of Science and Technology, Thuwal, 23955-6900 Saudi Arabia; 4 Computer, Electrical and Mathematical Sciences and Engineering Division, King Abdullah University of Science and Technology, Thuwal, 23955-6900 Saudi Arabia

## Abstract

Microtubules are highly dynamic structures that form spindle fibres during mitosis and are one of the most validated cancer targets. The success of drugs targeting microtubules, however, is often limited by the development of multidrug resistance. Here we describe the discovery and characterization of SSE15206, a pyrazolinethioamide derivative [3-phenyl-5-(3,4,5-trimethoxyphenyl)-4,5-dihydro-1H-pyrazole-1-carbothioamide] that has potent antiproliferative activities in cancer cell lines of different origins and overcomes resistance to microtubule-targeting agents. Treatment of cells with SSE15206 causes aberrant mitosis resulting in G2/M arrest due to incomplete spindle formation, a phenotype often associated with drugs that interfere with microtubule dynamics. SSE15206 inhibits microtubule polymerization both in biochemical and cellular assays by binding to colchicine site in tubulin as shown by docking and competition studies. Prolonged treatment of cells with the compound results in apoptotic cell death [increased Poly (ADP-ribose) polymerase cleavage and Annexin V/PI staining] accompanied by p53 induction. More importantly, we demonstrate that SSE15206 is able to overcome resistance to chemotherapeutic drugs in different cancer cell lines including multidrug-resistant KB-V1 and A2780-Pac-Res cell lines overexpressing *MDR-1*, making it a promising hit for the lead optimization studies to target multidrug resistance.

## Introduction

Microtubules are filamentous polymers of α- and β-tubulin heterodimers that provide structural integrity to eukaryotic cells. Several key cellular processes such as vesicular trafficking, intracellular signalling, cell motility and mitotic cell division depend on microtubules^1,2^. Importantly, microtubules form highly dynamic spindle fibres that are required for faithful segregation of duplicated chromosomes during mitosis^3,4^. Disruption of microtubule dynamics results in failure of cells to complete mitosis, which ultimately leads to apoptotic cell death^5,6^. Targeting microtubules, therefore, has been among the most successful and extensively deployed strategies for cancer therapeutics^2,7^.

Microtubule-targeting agents (MTAs) constitute a large group of chemically diverse compounds that can be divided into two classes based on their ability to affect microtubule polymerization. The stabilizing agents such as taxanes increase microtubule polymerization while destabilizing agents such as vinca alkaloids decrease it^7^. Many of these drugs are widely used in the clinic for treatment of various types of cancers with taxanes being particularly effective in solid tumours while vinca alkaloids showing efficacy in haematological malignancies^7^. However, despite the success, their wide-ranging clinical usage remains limited due to severe side effects and acquired resistance^2,8–10^. Overexpression of P-glycoprotein (Pgp) efflux pump, encoded by the *MDR-1* gene, is the most common mechanism involved in the development of resistance to several MTAs^11–13^ including vincristine, vinblastine, taxol, and docetaxel^11^. Point mutations in tubulin that affect its drug binding and altered expression of β-III tubulin isoform can also result in decreased sensitivity to MTAs^14–16^. Development of new MTAs can, therefore, help in overcoming resistance, improving tumour selectivity, and reducing the side effects^7^. New MTAs such as epothilones have been shown to overcome taxane resistance in clinical trials^17^. An epothilone derivative, ixabepilone has been approved by Food and Drug Administration (FDA) for the treatment of drug-resistant metastatic breast cancer^8,18^. Similarly, several preclinical studies have reported new MTAs that are able to overcome drug resistance^19–21^.

Compounds with heterocyclic pyrazoline ring are widely present in various natural products^22–27^ and are known to possess anti-tumor^28,29^, anti-angiogenic^30^, and anti-inflammatory^31^ properties. Some of these compounds are also known to inhibit microtubule assembly. 1-methyl-1H-indole–pyrazoline hybrids^32^ and 1-(30,40,50-trimethoxybenzoyl)-3,5-diarylpyrazoline scaffolds^33^, for example, have been reported to inhibit tubulin polymerization in the sub-micromolar to micromolar range. Similarly, N-benzoylated pyrazolines^34^ inhibited microtubule polymerization at nano-molar concentrations. We have recently synthesized and characterized the antiproliferative potential of novel compounds based on modified chalcones^35^. In continuation of our work we have prepared a small library of chalcone derivatives, pyrazolinethioamides, and have evaluated their antiproliferative potential. Furthermore, we have identified and characterized one of these compounds, SSE15206 [3-phenyl-5-(3,4,5-trimethoxyphenyl)-4,5-dihydro-1H-pyrazole-1-carbothioamide], as a microtubule polymerization inhibitor that overcomes multidrug resistance. We demonstrate its effects on cell proliferation, cell cycle regulation, tubulin polymerization and apoptosis. To our knowledge, this is the first study of the inhibition of tubulin polymerization by these compounds that incorporates their potential to overcome multidrug resistance.

## Results

### Several pyrazolinethioamide derivates have anti-proliferative activities against colorectal cancer cells

**Figure 1:**
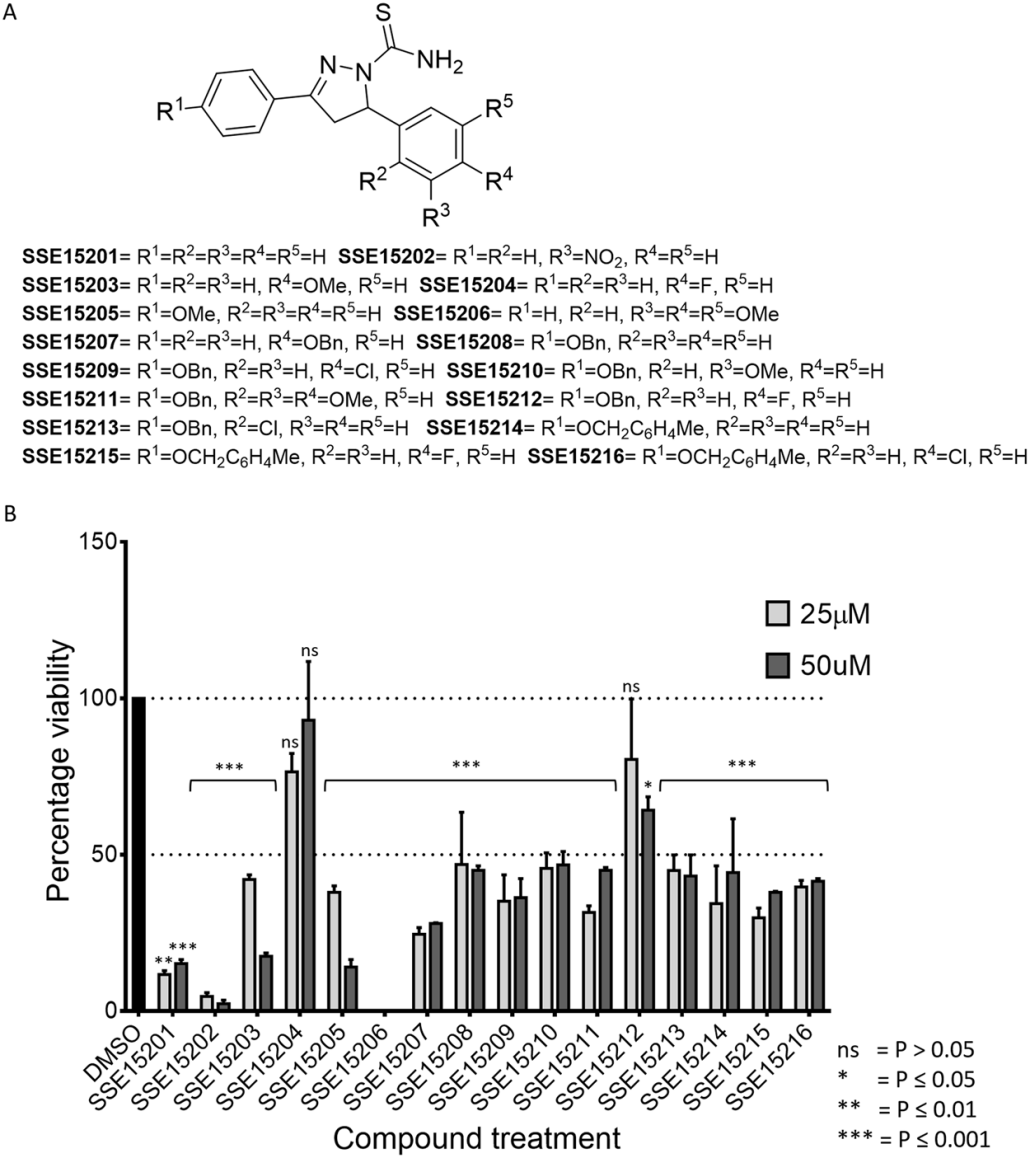
Structures and antiproliferative activities of pyrazolinethioamides (**A**). General structure of SSE152XX compound library. (**B**) Antiproliferative activities the SSE152XX compounds at 25 µM and 50 µM in HCT116 human colon cancer cell line. Cells were treated with two concentrations of the compounds for three days, followed by staining with SRB. Percentage inhibition was calculated with reference to the DMSO treated control cells. The graph represents results from two independent experiments done in duplicates.

An in-house library of 16 pyrazolinethioamide derivatives (SSE15201-SSE15216; Fig. 1A) was evaluated for antiproliferative activity against HCT116 colorectal cancer cells in a three-day SRB proliferation assay. Most compounds inhibited cell proliferation by more than 50% at 25 μM and 50 μM concentrations; only two compounds, SSE15204 and SSE15212, both fluorinated at the R^4^ position, failed to significantly inhibit proliferation at either concentration (Fig. 1). In the next step, half-maximal growth inhibitory (GI_50_) concentrations of all the compounds were determined. Eleven of the tested compounds had three-day GI_50_ values of less than 10 μM (Table 1A). Four compounds had GI_50_ values from 10 μM to 21 μM whereas, both fluorinated compounds were not active (GI_50_ > 50 μM). In general, the presence of an electronegative halogen atom (F, Cl) at R^4^ position and bulky OBn or MeC_6_H_4_CH_2_O group at R^1^ position led to lower activities. SSE15206, which contained three OMe groups at R^2^, R^3,^ and R^4^ positions was identified as the most potent compound with a GI_50_ value of 197 ± 0.05 nM in HCT116 cells (Table 1). SSE15206 also exhibited potent antiproliferative activities against several other cancer cell lines of different origins (leukaemia, breast, lung, and cervical) with sub-micromolar GI_50_ values for most of them (Table 1B).

**Table 1:** *A*. GI_50_ values of compounds in relation to their structure, in a three-day SRB proliferation assay in HCT116 cells. Values are presented as an average of at least three independent experiment together with standard deviation

Compound	R^1^	R^2^	R^3^	R^4^	R^5^	GI_50_ values (µM) ± SD	n
**A**							
SSE15201	H	H	H	H	H	5.76 ± 0.18	4
SSE15202	H	H	NO_2_	H	H	3.26 ± 0.11	4
SSE15203	H	H	H	MeO	H	5.02 ± 0.12	4
SSE15204	H	H	H	F	H	>50	4
SSE15205	MeO	H	H	H	H	8.69 ± 0.37	4
SSE15206	H	H	MeO	MeO	MeO	0.197 ± 0.05	3
SSE15207	H	H	H	BnO	H	1.30 ± 0.13	4
SSE15208	BnO	H	H	H	H	7.76 ± 0.19	4
SSE15209	BnO	H	H	Cl	H	20.55 ± 2.04	4
SSE15210	BnO	H	MeO	H	H	13.87 ± 0.19	4
SSE15211	BnO	H	H	MeO	H	8.71 ± 0.31	4
SSE15212	BnO	H	H	F	H	>50	4
SSE15213	BnO	Cl	H	H	H	12.19 ± 0.14	4
SSE15214	MeC_6_H_4_CH_2_O	H	H	H	H	6.98 ± 0.13	4
SSE15215	MeC_6_H_4_CH_2_O	H	H	F	H	9.88 ± 0.11	4
SSE15216	MeC_6_H_4_CH_2_O	H	H	Cl	H	13.94 ± 0.05	4
**B**							
**Cancer Type**		**Cell line**				**GI**_**50**_ **values (µM) ± SD**	
Breast		BT-549				0.445 ± 0.298 (n = 2)	
		CAL-51				0.286 ± 0.15 (n = 2)	
		MDA-MB-231				0.756 ± 0.077 (n = 3)	
Leukemia		MOLM-13				0.264 ± 0.046 (n = 2)	
		MV-4-11				0.362 ± 0.037 (n = 2)	
		K562				0.776 ± 0.467 (n = 2)	
Colorectal		HCT116				0.197 ± 0.049 (n = 3)	
		DLD-1				0.505 ± 0.239 (n = 4)	
		HCT-15				0.145 ± 0.046 (n = 3)	
Lung		A549				1.2692 ± 0.401 (n = 2)	
Cervical		HeLa				0.173 ± 0.031 (n = 3)	
Ovarian		A2780				0.145 ± 0.006 (n = 2)	

*B*. GI_50_ values of the most potent compound, SSE15206 in cancer cell lines of different origins in a three-day SRB proliferation assay.

### SSE15206, a pyrazolinethioamide derivative, induces mitotic arrest

**Figure 2:**
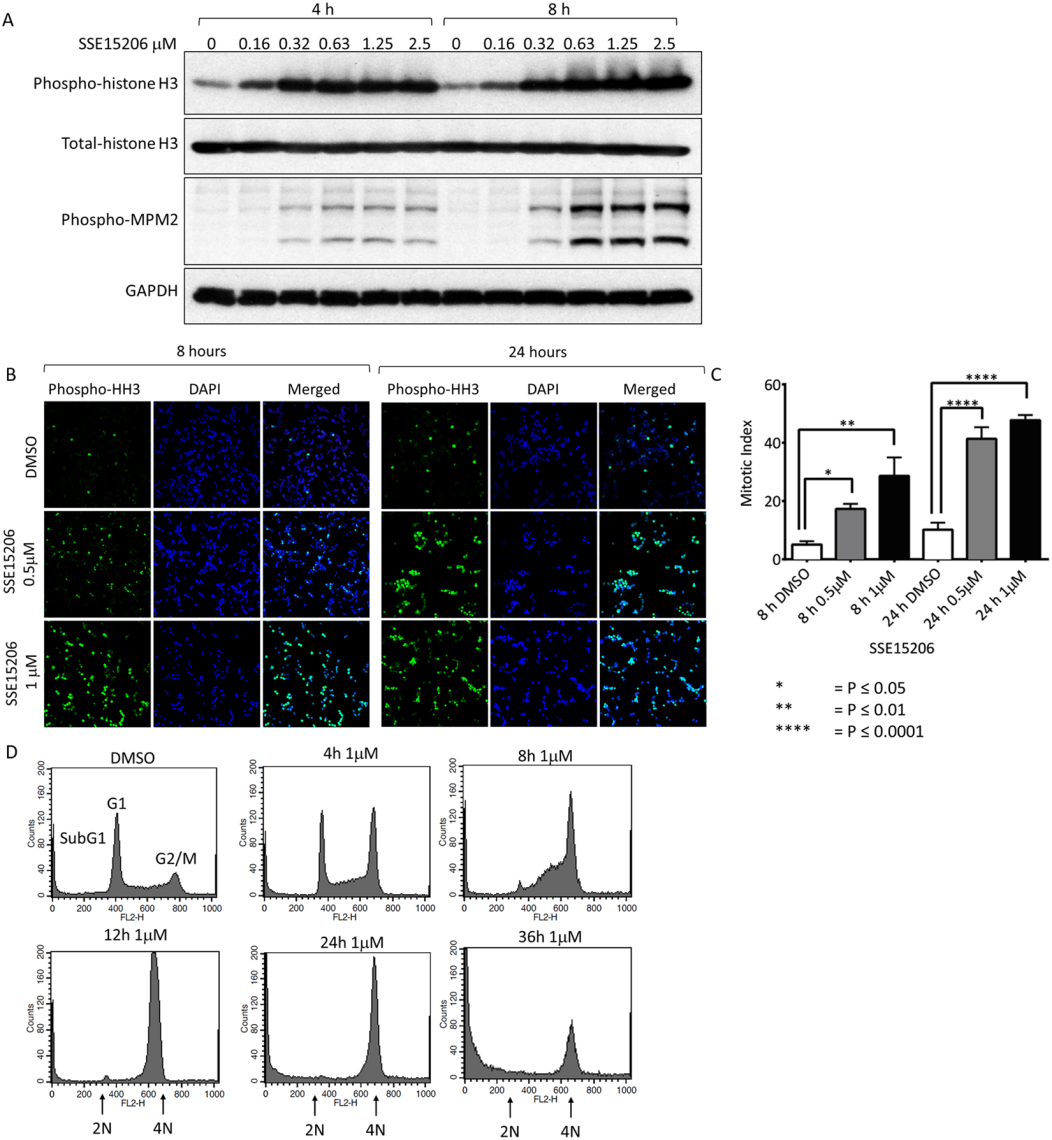
Induction of mitotic arrest by SSE15206. (**A**) Increase in histone H3 and MPM2 phosphorylation by SSE15206. HCT116 cells were treated with 0.5 μM, 1 μM and 2 μM SSE15206 for 4 and 8 hours. Phosphorylation of histone H3 (S10) and P-MPM2 was determined by western blotting; total-histone H3 and GAPDH were used as loading controls. (**B**) Immunofluorescence analysis for mitotic induction by SSE15206. HCT116 cells were treated with 0.5 μM and 1 μM SSE15206 for 8 and 24 hours and analyzed by immunofluorescence using phospho-histone H3 (S10) antibodies and DAPI. (**C**) Mitotic index (percentage mitotic cells) was calculated by taking the ratio of mitotic cells (Phospho-histone H3 positive, green) and total number of cells (DAPI positive, blue). Statistical significance was calculated by one-way ANOVA (one-way ANOVA 8 h: F (2,15) = 9.411, p < 0.05; post-hoc: 0.5 μM vs. DMSO *p < 0.05, 1 μM vs. DMSO **p < 0.01; one-way ANOVA 24 h: F (2,15) = 48.53, p < 0.0001; post-hoc: 0.5 μM vs. DMSO ****p < 0.0001, 1 μM vs. DMSO ****p < 0.0001). (**D**) SSE15206 induces cell cycle arrest at G2/M. HCT116 cells were analyzed through FACS following treatment with 1 μM SSE15206 for 4, 8, 12, 24 and 36 hours.

Treatment of cells with SSE15206 resulted in rounding up of cells which prompted us to look for induction of mitotic arrest using phosphorylation of histone H3 (S10) and MPM2 as mitotic markers. Indeed, dose-dependent increase in phosphorylation of both histone H3 and MPM2 was observed following treatment with SSE15206 for 4 and 8 hours indicating mitotic arrest. (Fig. 2A). Similarly, treatment with SSE15206 for 8 and 24 hours increased mitotic index (percentage mitotic cells) compared to the DMSO control as determined by immunofluorescence using phospho-histone H3 antibodies (Fig. 2B). The increase in the mitotic index was quantified and found to be statistically significant at both concentrations and time points (Fig. 2C). Activation of mitotic kinases, Aurora A, Aurora B and MPS1 in A549 cells accompanied mitotic arrest following SSE15206 treatment (Supp Fig. 2).

SSE15206 induced mitotic arrest in time- and dose-dependent manner, as determined through analysis of cell cycle profile in HCT116 cells. Within four hours of treatment with 1 μM drug, cells started accumulating in the G2/M phase of the cell cycle compared to the DMSO control (Fig. 2D). The G2/M arrest increased in a time-dependent manner and peaked at 12 hours. Treatment of cells for 24 and 36 hours, however, caused cells to accumulate in sub-G1 phase as well, indicating the onset of apoptosis (Fig. 2D). Similarly, SSE15206-induced dose-dependent increase in the G2/M arrest was accompanied by the increased sub-G1 population at higher doses of 1 μM and 2 μM (Supp Fig. 3).

### SSE15206 is a tubulin polymerization inhibitor

**Figure 3:**
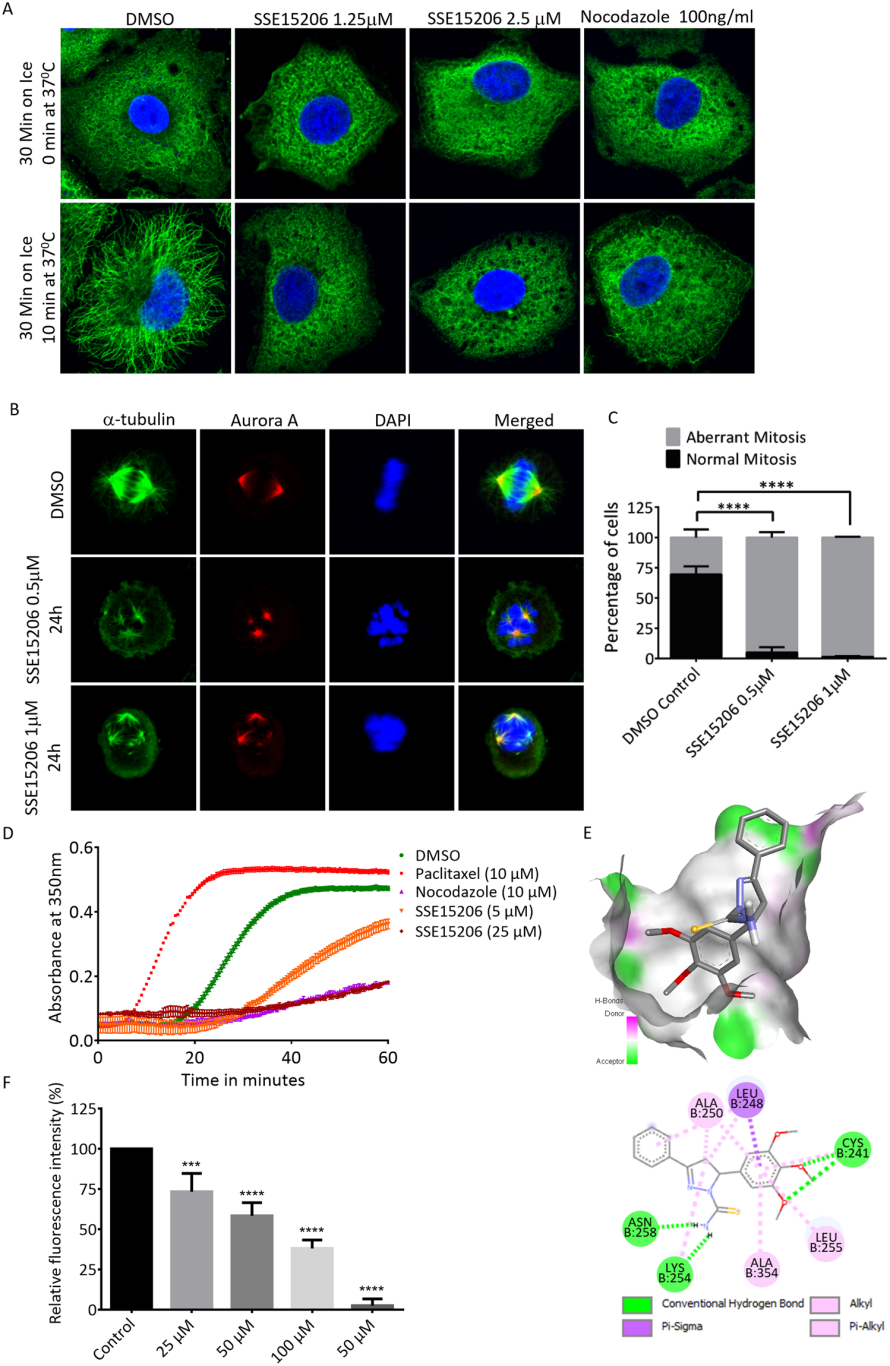
Inhibition of microtubule polymerization by SSE15206. (**A**) SSE15206 inhibits microtubule polymerization in cells. A549 cells were incubated on ice for 30 minutes followed by incubation at 37 °C for 10 minutes in the presence of 1.25 μM and 2.5 μM SSE15206 or DMSO control. 100 ng/ml (0.33 μM) nocodazole was used as a control for microtubule depolymerization. Cells were analyzed by immunofluorescence using alpha-tubulin antibodies (green) and DAPI (blue). (**B**) SSE15206 induces the formation of aberrant mitotic spindles. A549 cells were treated with 0.5 μM and 1 μM SSE15206 for 24 hours, fixed and stained for Aurora-A (red), tubulin (green) and DNA (blue). (**C**) Increased aberrant mitotic spindles upon treatment with 0.5 and 1 μM SSE15206 (one-way ANOVA: F (1,4) = 246.8, p < 0.0001; post-hoc: SSE15206 0.5 μM vs. DMSO ****p < 0.0001, SSE15206 1 μM vs. DMSO ****p < 0.0001) (**D**)*. In vitro* inhibition of tubulin polymerization by SSE15206. Polymerization of purified tubulin was measured in the presence of 5 μM and 25 μM SSE15206. DMSO was used as a solvent control while paclitaxel and nocodazole were used as controls for tubulin polymerization and depolymerization, respectively. (**E**) Docking of SSE15206 in the colchicine binding site of tubulin by Auto Dock Vina (upper panel). H-bonding interaction of SSE15206 with the beta subunit of tubulin in the docked structure (lower panel). (**F**) SSE15206 displaces colchicine in the competition assay. Different concentrations of SSE15206 were incubated with tubulin in the presence of 20 μM colchicine followed by measurement of fluorescence intensity. Colchicine displacement was quantified by relative fluorescent intensity (expressed as %, one-way ANOVA: F (4,10) = 84.5, p < 0.0001; post-hoc 25 μM vs. Control ***p < 0.001; 50 μM vs. Control ****p < 0.0001, 100 μM vs. Control ****p < 0.0001, 50 μM****p < 0.0001).

Induction of mitotic arrest is often a consequence of perturbation of microtubule dynamics by MTAs^2^. We, therefore, investigated whether SSE15206 could interfere with microtubule dynamics in A549 lung carcinoma cell line using an immunofluorescence-based microtubule repolymerization assay^36^. Treatment with 1.25 μM and 2.5 μM concentrations of SSE15206 resulted in the failure of microtubules to repolymerize at 37 °C following incubation on ice, similar to the positive control nocodazole (Fig. 3A). Microtubules are most dynamic during mitosis and treatment with microtubule depolymerization inhibitors results in failure of cells to form a functioning mitotic spindle. SSE15206-arrested mitotic cells had defective spindles and misaligned chromosomes compared to the mitotic cells in DMSO-treated controls (Fig. 3B). This increase in cells with aberrant mitotic spindles and misaligned chromosomes was statistically significant (Fig. 3C).

An *in-vitro* assay with purified tubulin confirmed that SSE15206 bound and directly inhibited tubulin polymerization at both tested concentrations (5 μM and 25 μM; Fig. 3D). At 25 μM, SSE15206 completely inhibited tubulin polymerization which was comparable to inhibition by 10 μM nocodazole, a positive control for tubulin depolymerization (Fig. 3D). Many microtubule polymerization inhibitors bind to the colchicine binding site of tubulin, one of the three major binding sites in tubulin for MTAs^2,37^. We, therefore, performed docking studies that predicted a favourable interaction between SSE15206 and colchicine binding site of tubulin (Fig. 3E). Verification of docking algorithms was provided by correctly placed colchicine in its binding site (Supp. Figure 4A). The overlap of colchicine with SSE15206 shows that the trimethoxy phenyl ring shared by the two ligands interacts with the same binding site (Supp. Figure 4B). The docking also predicted H-bond interaction between thioamide moiety of the inhibitor and Cys254 and Asn258 of the beta subunit of tubulin (Fig. 3E lower panel).

The docking predictions for SSE15206 binding to colchicine site were validated through fluorescent-based colchicine displacement assay^38^. SSE15206 caused a dose-dependent significant decrease in fluorescence signal indicating colchicine displacement from tubulin (Fig. 3E). Nocodazole, a known colchicine site binder also displaced colchicine as indicated by a reduction in fluorescent signal (Fig. 3F).

### SSE15206 induces p53 and apoptosis in different cancer cell lines

**Figure 4:**
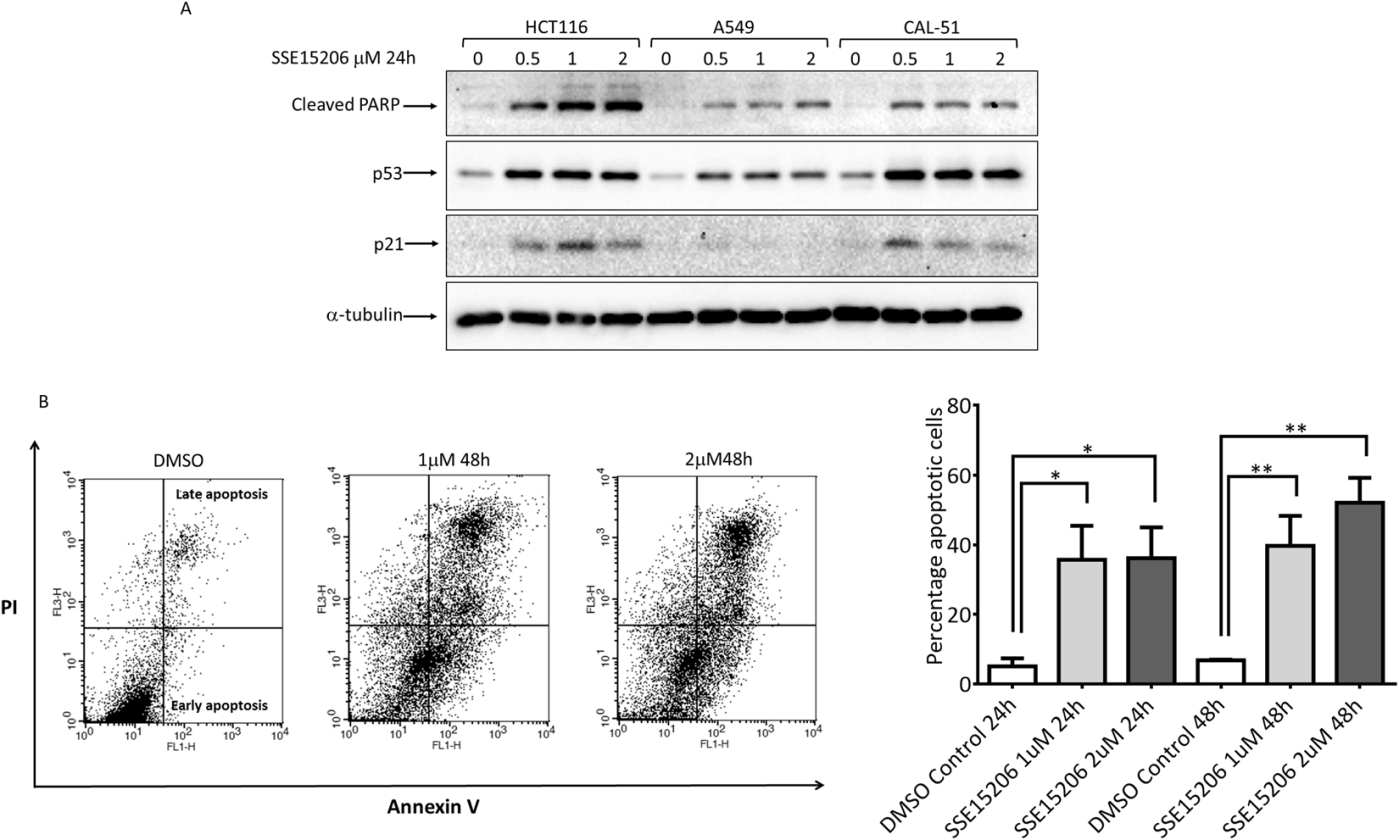
Induction of apoptosis by SSE15206. (**A**) SSE15206 induces cleaved PARP and p53. HCT116, A549, and CAL-51 cells were treated with 0.5 μM, 1 μM and 2 μM SSE15206 for 24 hours and levels of cleaved PARP, p53, and p21 were analyzed by western blotting using specific antibodies. Alpha-tubulin was used as loading control. (**B**) Increased Annexin V/PI staining in cells treated with SSE15206. Cells treated with 1 μM and 2 μM SSE15206 for 24 and 48 hours were stained with FITC-labelled Annexin V/PI and analyzed by FACS. The left panel shows FACS data for cells treated for 48 hours, whereas the graph on the right shows quantification of cells treated with two concentrations of SSE15206 for 24 and 48 hours (One-way ANOVA 24 h: F (2,6) = 5.309, p < 0.05; post-hoc: SSE15206 1 μM vs. DMSO *p < 0.05, SSE15206 2 μM vs. DMSO *p < 0.05; One-way ANOVA 48 h: F (2, 6) = 13.05, p < 0.01; post-hoc SSE15206 1 μM vs. DMSO **p < 0.01, SSE15206 2 μM vs. DMSO **p < 0.01).

Disruption of microtubule dynamics results in enhanced phosphorylation and stabilization of p53 protein accompanied by increased expression of p21^39^. Treatment of three different cell types, HCT116, A549 and CAL-51 with SSE15206 for 24 hours, resulted in increased expression of p53 and p21 compared to DMSO control. (Fig. 4A).

Increased cleavage of Poly (ADP-ribose) polymerase (PARP), an apoptotic marker was also seen in these cells (Fig. 4A, upper panel). The induction of apoptosis was further confirmed by Annexin V/PI staining followed by FACS analysis. Treatment of cells with 1 μM and 2 μM SSE15206 for 24 and 48 hours resulted in significant increase in apoptosis at both concentrations and time points (Fig. 4B). FACS results on the left show increased apoptosis (late and early) at both concentrations following 48-hour treatment. Quantification and statistical significance are shown in the graph on the right (Fig. 4B).

### SSE15206 overcomes multidrug resistance *in vitro*

**Figure 5:**
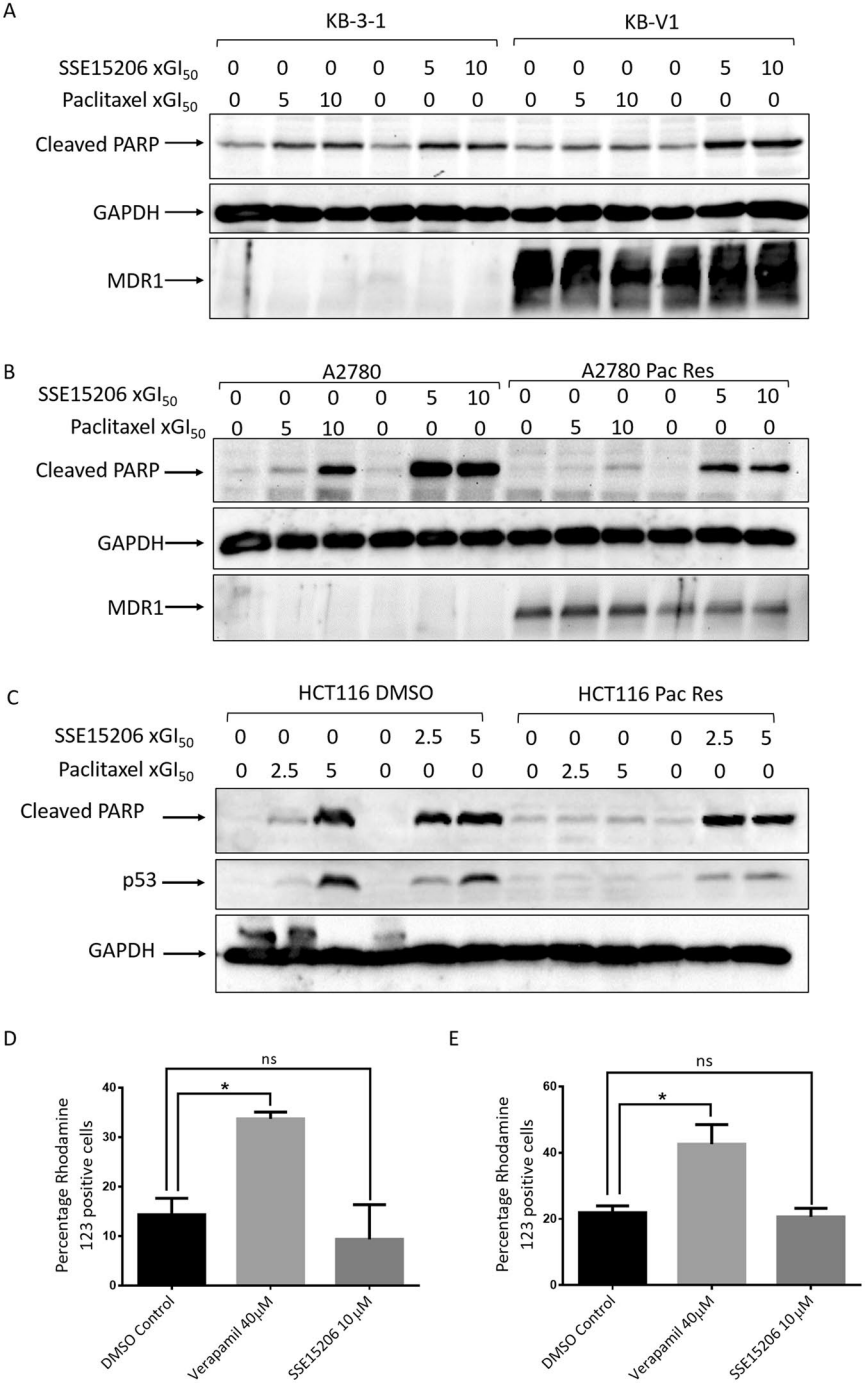
SSE15206 causes apoptosis in drug-resistant cell lines. (**A** and **B**) SSE15206 induces PARP cleavage in control and multidrug-resistant cell lines. Both cell line pairs, KB-3–1/KB-V1 and A2780/A2780-Pac-Res were treated with 5× and 10 × GI_50_ values of SSE15206 or paclitaxel for 24 hours followed by analysis of cleaved-PARP through western blotting. Blots for GAPDH and MDR-1 show loading control and MDR-1 overexpression, respectively in KB-V1 and A2780-Pac-Res cell lines. (**C**) PARP cleavage and p53 induction by SSE15206 and paclitaxel in HCT116 DMSO and HCT116-Pac-Res cell lines. Cells were treated with 2.5× and 5 × GI_50_ values of SSE15206 or paclitaxel for 24 hours followed by analysis of PARP cleavage and p53 induction. Levels of GAPDH were used as a loading control. *D and E*. Effect of 40 μM Verapamil (positive control for inhibition of Pgp) and 10 μM SSE15206 on the efflux of Rhodamine 123 by KB-V1 (**D**) and A2780-Pac-Res (**E**) cells. (One-way ANOVA; n.s. = not significant, *p ≤ 0.05). (One-way ANOVA *D*: F (2, 3) = 19.81, p < 0.05; post-hoc: SSE15206 10 μM vs. DMSO p > 0.05, Verapamil 40 μM vs. DMSO *p < 0.05; One-way ANOVA *E*: F (2, 3) = 15.99, p < 0.05; post-hoc SSE15206 10 μM vs. DMSO p > 0.05, Verapamil 40 μM vs. DMSO *p < 0.05).

Overexpression of Pgp efflux pumps is the most common mechanism of acquired resistance to MTAs. To determine whether SSE15206 can overcome multidrug resistance, we evaluated its efficacy in different drug-resistant cell lines including KB-V1, a multidrug resistant cell line that overexpresses *MDR-1*^40^. Compared to the parental KB-3–1 cells, KB-V1 were highly resistant to paclitaxel (>1000-fold), and vinblastine (89-fold) but sensitive to SSE15206 (0.94-fold; Table 2). Similar results were obtained in another *MDR-1* overexpressing multidrug resistant cell line A2780-Pac-Res, which was highly resistant to paclitaxel (101-fold), vincristine (16-fold), and etoposide (56-fold) but not to SSSE15206 (1.54-fold) when compared with the parental A2780 cell line (Table 2). The efficacy of SSE15206 in two MDR-1 overexpressing cell lines indicates that it is not a substrate of Pgp efflux pump and therefore can overcome multidrug resistance. Moreover, SSE15206 caused apoptotic cell death in both parental and *MDR-1* overexpressing cell lines as indicated by increased PARP cleavage, in contrast to paclitaxel which did so only in the parental cells (Fig. 5A and B).

**Table 2:** SSE15206 overcomes multidrug resistance.

Cell line	Compound GI_50_				
	Paclitaxel (nM)	SSE15206 (nM)	Vinblastine (nM)	Vincristine (nM)	Etoposide (μM)
KB-3-1 (Control)	0.85 ± 0.16 (n = 3)	378.33 ± 0.92 (n = 3)	0.89 ± 0.14 (n = 3)	ND	0.74 ± 0.025 (n = 2)
KB-V1 (MDR-1)	896.33 ± 65.62 (n = 3)	354 ± 89.82 (n = 3)	79.3 ± 103 (n = 3)	ND	16.07 ± 12.18 (n = 2)
**Fold Resistance**	**1054**	**0.94**	**89**		**21.72**
A2780 (Control)	2.03 ± 0.84 (n = 2)	145.65 ± 0.64 (n = 2)	ND	6.04 ± 1.59 (n = 4)	0.65 (n = 1)
A2780-Pac-Res	204.65 ± 3.18 (n = 2)	224.45 ± 21.29 (n = 2)	ND	95.1 ± 24.81 (n = 4)	36.17 (n = 1)
**Fold Resistance**	**101**	**1.54**		**15.7**	**56**
HCT116 (Control)	1.01 ± 0.026 (n = 3)	261.63 ± 143.22 (n = 3)	ND	ND	ND
HCT116-Pac-Res	178.67 ± 65.17 (n = 3)	316.53 ± 19.69 (n = 3)	ND	ND	ND
**Fold Resistance**	**176**	**1.2**			

Efficacy of different chemotherapeutic drugs and SSE15206 in different drug-resistant cell lines and their parental controls is shown. The GI_50_ values (in nM or μM) are presented as mean of (n) independent experiments ± standard deviation. The resistance of cell lines to various drugs is presented as fold-resistance compared to the parental control. ND = not determined.

SSE15206 also overcame resistance to paclitaxel in HCT116-Pac-Res cell line which is highly resistant to paclitaxel (176-fold resistant) without *MDR-1* overexpression (Table 2). This cell line was generated by continuous exposure to increasing concentrations of paclitaxel over a period of 8 months. SSE15206 inhibited proliferation of the resistant cells with GI_50_ values comparable to DMSO control cells (1.2-fold, Table 2). Similarly, SSE15206 induced PARP cleavage in both resistant and parental HCT116 cell lines in contrast to paclitaxel which caused apoptotic cell death only in the parental cell line (Fig. 5C). Regulation of p53 in HCT116 cells also followed the trend seen for induction of cleaved-PARP (Fig. 5C).

We next evaluated the mechanism through which SSE15206 overcame multidrug resistance in KB-V1 and A2780-Pac-Res cell lines using rhodamine 123 efflux assay^41,42^. Efflux of loaded rhodamine 123 was significantly inhibited by 40 μM verapamil but not 10 μM SSE15206 in both the cell lines (Fig. 5D and E). This indicates that SSE15206 overcomes multidrug resistance in MDR-1 expressing cell lines because it is not a substrate of Pgp.

## Discussion

Despite the demonstrated anti-tumour activity of MTAs against several cancer types, their wide-spread use and effectiveness are often limited by inherent and acquired drug resistance^8,11^. Notably, acquisition of resistance to one type of drug often causes resistance to several drug types or “multidrug resistance”, rendering various treatment options ineffective. Here we describe the synthesis and characterization of SSE15206 as a microtubule polymerization inhibitor that can overcome drug resistance, including multidrug resistance, to MTAs. SSE15206 inhibits microtubule polymerization to induce apoptosis in various cancer cells. Importantly, it inhibits proliferation of paclitaxel-resistant cells, including *MDR-1* expressing multidrug resistant cells, indicating that it is not affected by P-glycoprotein (Pgp).

Anti-proliferative effects of SSE15206 were related to increased phosphorylation of mitotic markers, histone H3, and MPM2^43^, indicating mitotic arrest which was accompanied by aberrant mitotic spindles and misaligned chromosomes (Fig. 2A and B). Cell cycle analysis further confirmed G2/M arrest following SSE15206 treatment in a time- and dose-dependent manner. These effects were associated with the ability of SSE15206 to inhibit microtubules polymerization as confirmed by cell-based microtubule repolymerization and *in vitro* tubulin polymerization assays. Interestingly, the anti-proliferative effects of SSE15206 are obvious in the sub-micromolar range for many cell types, which is considerably lower than the concentration required (25 μM) for complete depolymerization in the *in vitro* assays. Indeed, many MTAs inhibit microtubule dynamics at concentrations 10–100 fold lower than the concentration required to affect the total polymer mass^2,44^.

Microtubule depolymerizing drugs mostly bind to tubulin on either the v*inca* or the colchicine sites^7^. Many of the colchicine site binders have a trimethoxy phenyl group similar to the one present in SSE15206^37^. Docking studies predicted SSE15206 binding into the colchicine binding site of tubulin. The trimethoxy group of SSE15206 was located in the β-tubulin unit in the vicinity of amino acid residue Cys241 similar to the interaction of a trimethoxy group of colchicine in the binding cavity. Furthermore, the thioamide moiety of the compound made H-bond interactions with the amino acids Lys254 and Asn258 of the β-tubulin (Fig. 3E lower panel). Dose-dependent displacement of colchicine by SSE15206 validated these docking predictions (Fig. 3F). Several structurally diverse, colchicine binding site inhibitors (CBSI) currently under investigation show promising anti-cancer activities^37^. Although the narrow therapeutic index has hampered their development as anti-cancer drugs, new CBSIs are still being pursued owing to their ability to overcome Pgp- and β-III tubulin-mediated drug resistance^37^.

Development of multidrug resistance is a major limiting factor in the efficacy of MTAs^11,12^. The most frequent mechanism employed by tumours to acquire multidrug resistance is overexpression of efflux pumps, such as Pgps of the ABC transporter family^14,45^. The Pgp proteins actively pump drugs out of the cells and many routinely used MTAs are their substrates. SSE15206 inhibited proliferation of Pgp overexpressing KB-V1 and A2780-Pac-Res cell lines at GI_50_ values comparable to the corresponding parental control cell lines (Table 2). This suggests that SSE15206 either directly inhibits Pgp or is not a Pgp substrate and thereby can effectively overcome multidrug resistance in cells with *MDR-1* overexpression. Efflux assay using rhodamine 123 ruled out the direct inhibition of Pgp and thereby supported the latter hypothesis that SSE15206 is not a Pgp substrate. Although colchicine itself is a known Pgp substrate, several CBSI can either inhibit Pgp or are poor Pgp substrates^46,47^. SSE15206 also overcame resistance to paclitaxel in HCT116-Pac-Res cells that are highly resistant to paclitaxel (Table 2) without overexpression of *MDR-1* or β-III tubulin (Unpublished data). The mechanism of resistance of these cells is currently under investigation, but mutations in β-tubulin that affect binding of paclitaxel may be the most likely mechanism^14,16^.

The effectiveness of MTAs is mainly dependent on their ability to induce apoptotic cell death through the mitochondrial pathway^48^. The efficacy of SSE15206 also relies on its ability to induce apoptosis. Treatment with SSE15206 resulted in induction of p53 and increased PARP cleavage as well as significant increase in Annexin V/PI staining, all indicating onset of apoptotic cell death. Although robust p53 induction is seen in three cell lines tested (Fig. 4A), SSE15206 potently inhibits proliferation of several cell lines with mutant p53 (Table 1) including the isogenic p53 wild-type and mutant HCT116 cells (data not shown). This is in accordance with reports that some MTAs can induce apoptotic cell death independent of p53 function through alternative mechanisms including upregulation of pro-apoptotic proteins^49–51^. The reduced efficacy of paclitaxel in multidrug-resistant cell lines compared to parental cells correlated with its inability to induce apoptotic cell death in these cells (PARP cleavage; Fig. 5A and B). SSE15206 however, was able to induce apoptosis in cells irrespective of *MDR-1* overexpression. Similarly, SSE15206 induced apoptosis in HCT116-Pac-Res and parental cells in contrast to paclitaxel, which induced apoptosis in the later cell line only (Fig. 5C). In conclusion, we have described the discovery and characterization of SSE15206 as a potent microtubule polymerization inhibitor that overcomes resistance to other MTAs including multidrug resistance. This inhibitor will serve as a lead compound for further optimization for improved potency and evaluation of *in vivo* efficacy in multidrug-resistant cell lines.

## Methods

### Chemical library

**Figure 6:**
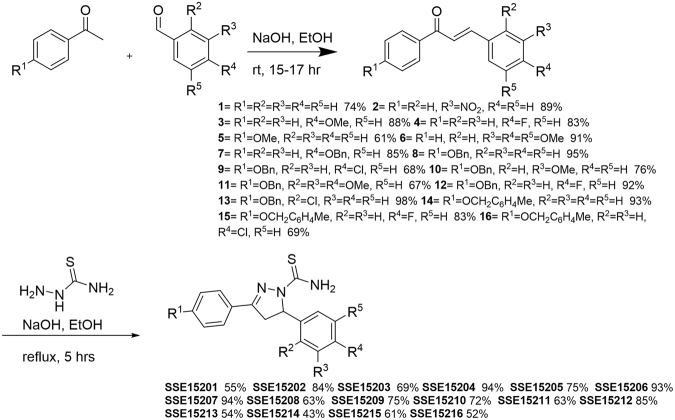
General procedure for the synthesis of the compound library.

An in-house chemical library, comprising of 16 compounds was built using a two-step synthesis protocol and involved Claisen-Schmidt reaction and condensation of resulting chalcone with thiosemicarbazide (as shown in Fig. 6 and supplementary information).

### Antibodies and reagents

Antibodies against phospho-histone-H3 (S10), alpha-tubulin, Phospho-MPM2, and GAPDH were purchased from MerckMillipore. Antibodies for cleaved-PARP, phospho-Aurora A/B/C, and total-Aurora A were from Cell Signaling. Monoclonal p53 and p21 antibodies were purchased from ThermoFisher and BD Biosciences, respectively. The MDR-1 antibody was obtained from Santa Cruz. HRP-labeled anti-mouse and anti-rabbit secondary antibodies were purchased from Dako. Fluorescently-labelled anti-mouse and anti-rabbit secondary antibodies were from ThermoFisher.

### Cell culture and proliferation assays

Cell lines were maintained in their recommended culture medium (DMEM or RPMI) supplemented with 10% FBS and 1 × Antibiotic-Antimycotic at 37 °C in humified incubators with 5% CO_2_. All the cells used were passaged for less than 6 months before replacement from early passage frozen stocks. KB-3–1 and KB-V1 were generously provided by Professor Michael Gottesman (Centre for Cancer Research, NCI). A2780 control and A2780-Pac-Res cell lines were from Professor Spiros Linardopoulos (Institute of Cancer Research UK). For adherent cells, Sulforhodamine B (SRB) assay was used to determine the effect of compounds on cell proliferation as described elsewhere^52^. Briefly, cells plated in 96-well plates were treated with three-fold dilutions of the compounds for 72 hours. Cells were then fixed with ice-cold TCA (3% final concentration) for 2 h at 4 °C and stained with 0.06% SRB. SRB bound to the stained cells was solubilized in 10 mM Tris pH 10.5. O.D. was measured at 490 nm using microplate reader (BioTek). GI_50_ values were calculated using GraphPad Prism. For non-adherent cells, MTS-based proliferation assay (CellTiter 96 Aqueous; Promega) was used according to the manufacturer’s instructions.

### Immunoblotting and Immunofluorescence

Cells were collected either in Triton X100-based lysis buffer (50 mmol/L NaCl, 25 mmol/L Tris-HCl, pH 7.5, 1% Triton X-100 supplemented with phosphatase and protease inhibitors) or 2 × SDS-sample buffer. Equal amounts of proteins were resolved using SDS-PAGE, transferred onto nitrocellulose membrane, blocked with 5% skimmed milk and incubated with primary antibodies at 4 °C overnight. Blots were washed with PBST (PBS + 0.1% Tween) and incubated with HRP-labelled secondary antibodies for 1 hour at room temperature. After further washing, blots were developed using enhanced chemiluminescence reagent on BioRad ChemiDoc system.

For Immunofluorescence, cells were grown on Poly-L-lysin coated coverslips in 6-well plates and treated with SSE15206 for concentrations and times indicated. At the end of treatments, cells were fixed and stained using α-tubulin (1:500) and Aurora A (1:100) antibodies as previously described^53^. Fluorescently labelled secondary antibodies were used at 1:500 dilution, while nuclei were stained with DAPI (1:1000). Images were taken with Nikon confocal microscope.

### Cell cycle and apoptosis analysis

For cell cycle analysis, cells were trypsinized and fixed in 85% ice-cold ethanol. Fixed cells were washed in PBS containing 1% FBS and stained with propidium iodide/RNase solution (10 μg/ml PI/0.5% RNase) for 30 minutes at 37 °C and analyzed using BD FACSCalibur. For Annexin V/PI apoptotic assay, treated cells were harvested and stained with Annexin V-FITC apoptosis detection kit (Sigma).

### Microtubule repolymerization assay

A549 cells were grown on Poly-L-lysin coated coverslips in 6-well tissue culture plates. Cells were incubated on ice for 30 minutes to depolymerize microtubules. Next, cells were treated with two different concentrations of the compound or DMSO and placed at 37 °C for 10 minutes. Cells were then fixed with ice-cold methanol and stained for immunofluorescence using alpha-tubulin antibodies as described above.

### *In vitro* Tubulin Polymerization Assay

Purified tubulin (50 µM) was incubated with different concentrations of compounds in the presence of 1 mM GTP in PIPES-based polymerization buffer (80 mM K-PIPES pH 6.8, 1 mM MgCl_2_, 1 mM EGTA) at 37 °C. Tubulin assembly was followed by turbidimetry variation at 350 nm every 30 sec during 1 h incubation. The experiment was performed in triplicates with paclitaxel and nocodazole as positive controls for tubulin polymerization and depolymerization, respectively. (This assay was performed at Ecrins Therapeutics in France)

### Molecular docking studies

Molecular docking for SSE15206 binding to the colchicine binding site of tubulin was performed using AutoDock Vina. The structure of SSE15206 was drawn using Chem3D, saved in pdb format and its energy was minimized using Avogadro. The reported 3D structure of tubulin consisting of the tubulin-DAMA-colchicine-stathmin-like domain complex was retrieved from the Protein Data Bank (PDB code: 1SA0). It was prepared for the docking studies by removing the stathmin-like domain, the C and D subunits and the ligands. The addition of hydrogen atoms was performed using MGLTools for AutoDock and the docking was performed using AutoDock Vina (Scripps Research Institute, USA)^54^. The docking was carried out using exhaustiveness of 8 and grid box dimensions of 20, 20, 20.

### Colchicine displacement assay

Fluorescence intensity of tubulin (4 μM) in the presence of colchicine (20 μM) and SSE15206 at indicated concentrations was measured. DMSO was used as a solvent control while 50 μM nocodazole was used as a positive control for colchicine displacement. Samples were incubated for 60 min at 37 °C before measurement of fluorescence (excitation at 355 nm and emission at 460 nm). (This assay was performed at Ecrins Therapeutics in France).

### Rhodamine 123 efflux assay

KB-V1 and A2780-Pac-Res cells were incubated with 5 μM rhodamine, for 1 hour at 37 °C in the presence or absence of inhibitors. Cells were then washed twice with PBS and incubated in the efflux medium (their respective growth media) in the presence of DMSO, 20 μM verapamil, and 10 μM SSE15206 for 3 hours. The percentage of rhodamine 123 positive cells was determined by Countess II FL Automated Fluorescent Cell Counter (Thermo Fisher Scientific). Experiments were done in duplicates with 4–5 readings for each sample in each experiment.

### Statistical analysis

For comparison between two groups, unpaired Student t-test was used, with additional Welsch correction in cases where the variances were unequal between the two groups. For data involving a comparison between more than two groups, one-way ANOVA was used. Time-dependent measurements were performed with one or two-way repeated measures ANOVA. Significant ANOVA analyses were followed by post-hoc pair-wise comparisons using Dunnett or Holm-Sidak post-hoc tests. Outliers were defined as values beyond two standard deviations from the group mean and were removed from the analyses. Significance was set at p < 0.05 for all tests and two-sided tests were performed. Statistical analysis was performed using GraphPad Prism version 7 and verified by SPSS version 23. All graphs were drawn with GraphPad Prism version 7.

## Electronic supplementary material

Supplementary Materials
