# Author Correction: Identification and characterization of SSE15206, a microtubule depolymerizing agent that overcomes multidrug resistance

**DOI:** 10.1038/s41598-018-24114-7

**Published:** 2018-04-18

**Authors:** Safia Manzoor, Aishah Bilal, Sardraz Khan, Rahim Ullah, Sunniya Iftikhar, Abdul-Hamid Emwas, Meshari Alazmi, Xin Gao, Ali Jawaid, Rahman Shah Zaib Saleem, Amir Faisal

**Affiliations:** 1 Department of Chemistry, Syed Babar Ali School of Science and Engineering, Lahore University of Management Sciences, Lahore, 54792 Pakistan; 2 Department of Biology, Syed Babar Ali School of Science and Engineering, Lahore University of Management Sciences, Lahore, 54792 Pakistan; 3 Imaging and Characterization Core Lab, King Abdullah University of Science and Technology, Thuwal, 23955-6900 Saudi Arabia; 4 Computer, Electrical and Mathematical Sciences and Engineering Division, King Abdullah University of Science and Technology, Thuwal, 23955-6900 Saudi Arabia

Correction to: *Scientific Reports* 10.1038/s41598-018-21642-0, published online 19 February 2018

The Article contains an error where the computation of statistical significance in Figure 1b is incorrect. The correct figure appears below as Figure 1.

**Figure 1:**
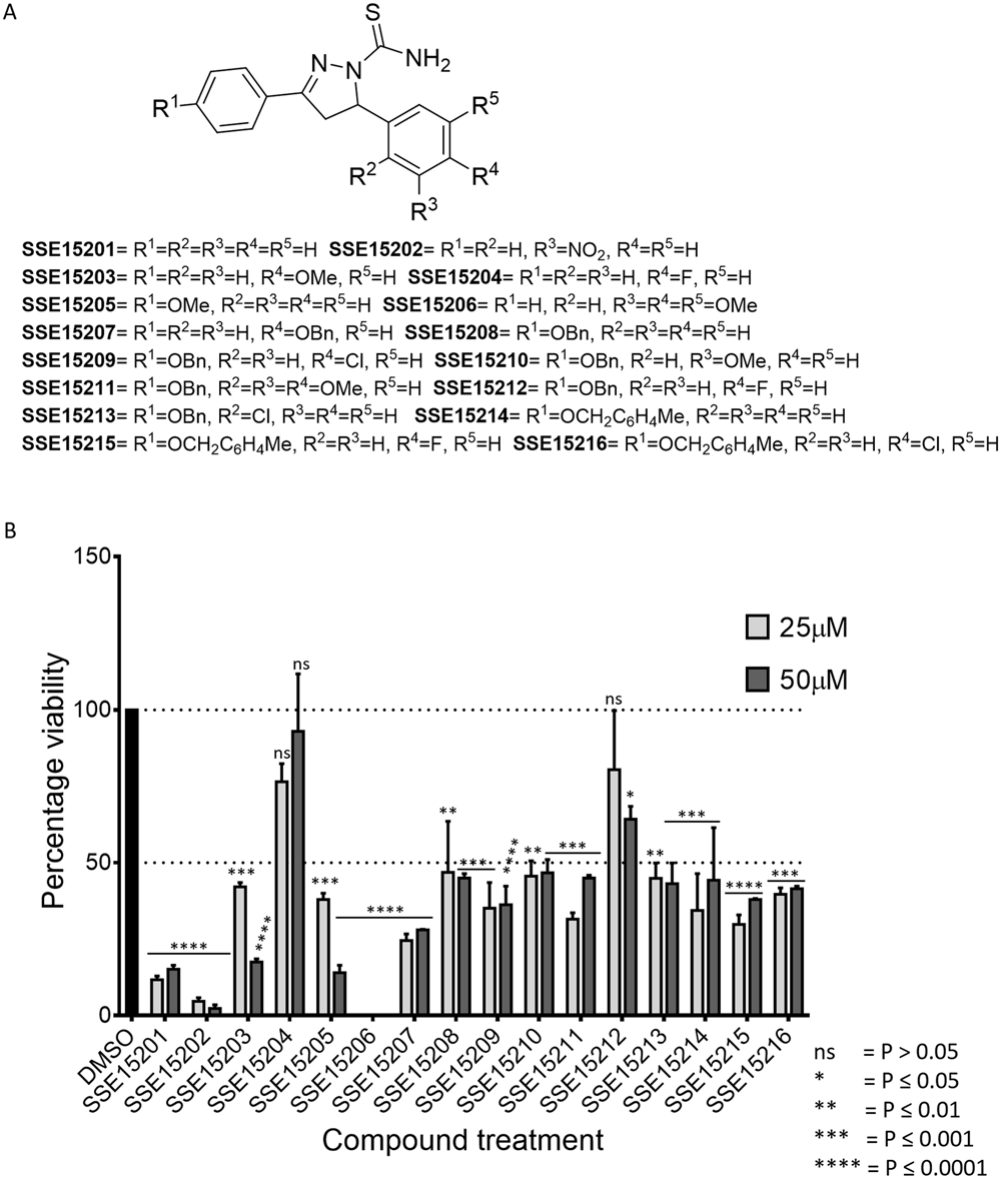
Structures and antiproliferative activities of pyrazolinethioamides (**A**). General structure of SSE152XX compound library. (**B**) Antiproliferative activities the SSE152XX compounds at 25 μM and 50 μM in HCT116 human colon cancer cell line. Cells were treated with two concentrations of the compounds for three days, followed by staining with SRB. Percentage inhibition was calculated with reference to the DMSO treated control cells. The graph represents results from two independent experiments done in duplicates.

