# Supplementary material for: Identification and characterization of SSE15206, a microtubule depolymerizing agent that overcomes multidrug resistance
Source: Sci Rep. 2018 Feb 19;8:3305. doi: 10.1038/s41598-018-21642-0 (PMC5818492; doi:10.1038/s41598-018-21642-0)
Supplement: Supplementary file 1 — Supplementary Materials [file 41598_2018_21642_MOESM1_ESM.pdf]

Identification and characterization of SSE15206, A microtubule depolymerizing agent that overcomes multidrug resistance.

Supplementary Materials:

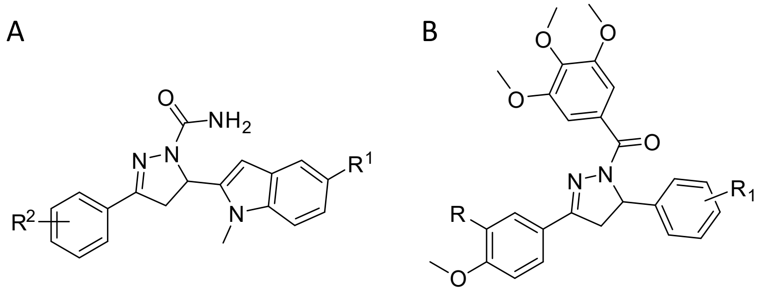

Supplementary Figure 1: General structures of 1-methyl-1H-indole–pyrazoline hybrids[1] (A) and 1-(30,40,50-trimethoxybenzoyl)-3,5-diarylpyrazolines[2] (B)

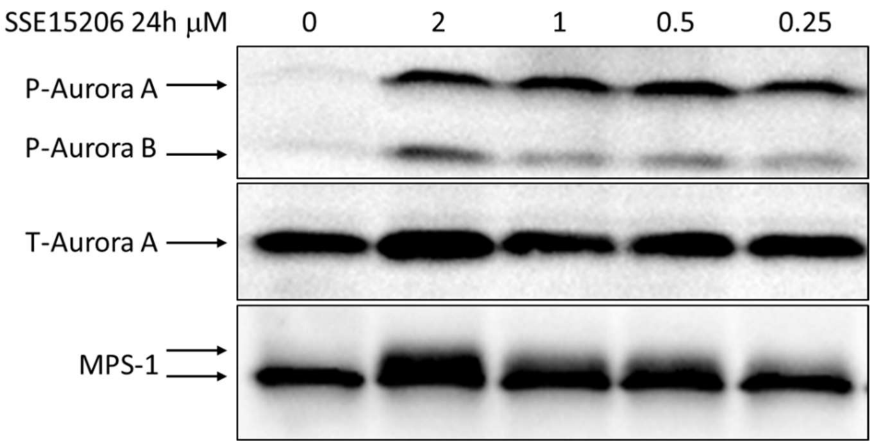

Supplementary Figure 2: Activation of mitotic kinases by SS15206 in A549 cells. Cells were treated with different concentrations of the inhibitor for 24h and analyzed by western blotting using specific antibodies. Activation of Aurora kinases A and B was measured using phospho-specific antibodies while MPS1 activation was measured by gel shift. Total Aurora A levels were used as loading control. Activation of these mitotic kinases is shown by increased phosphorylation of Aurora Kinases and reduced gel mobility of MPS1 kinase (a consequence of increased MPS1 phosphorylation).

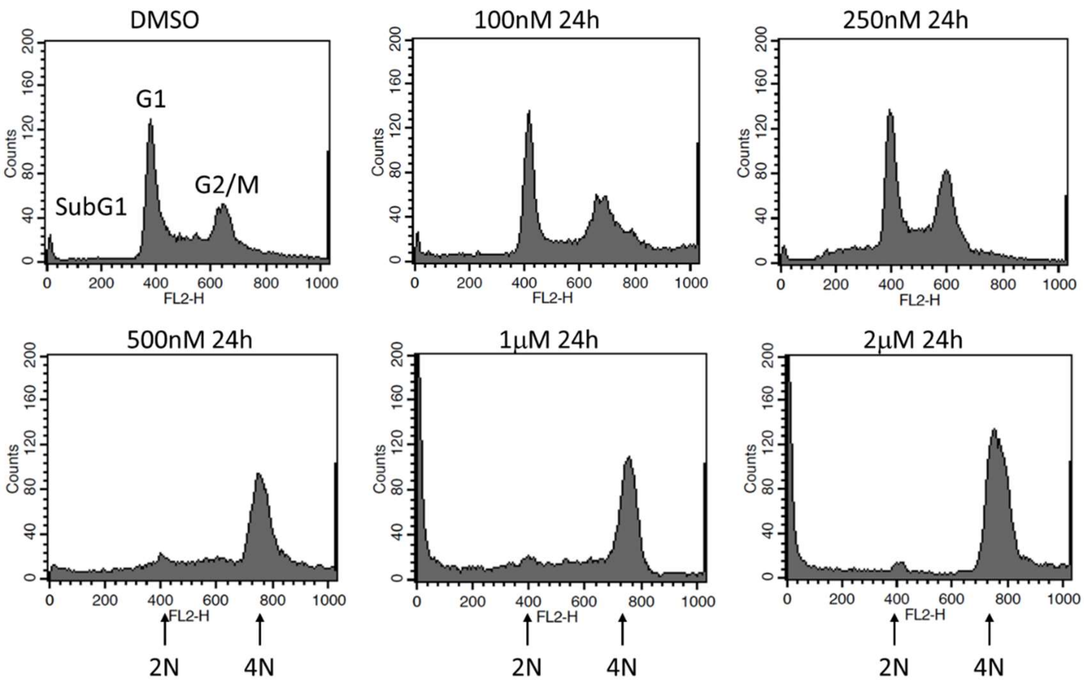

Supplementary Figure 3: SSE15206 induces cell cycle arrest at G2/M. HCT116 cells were treated with indicated concentration of SSE15206 for 24 hours. Cells were stained with Propidium Iodide and analyzed using FACS.

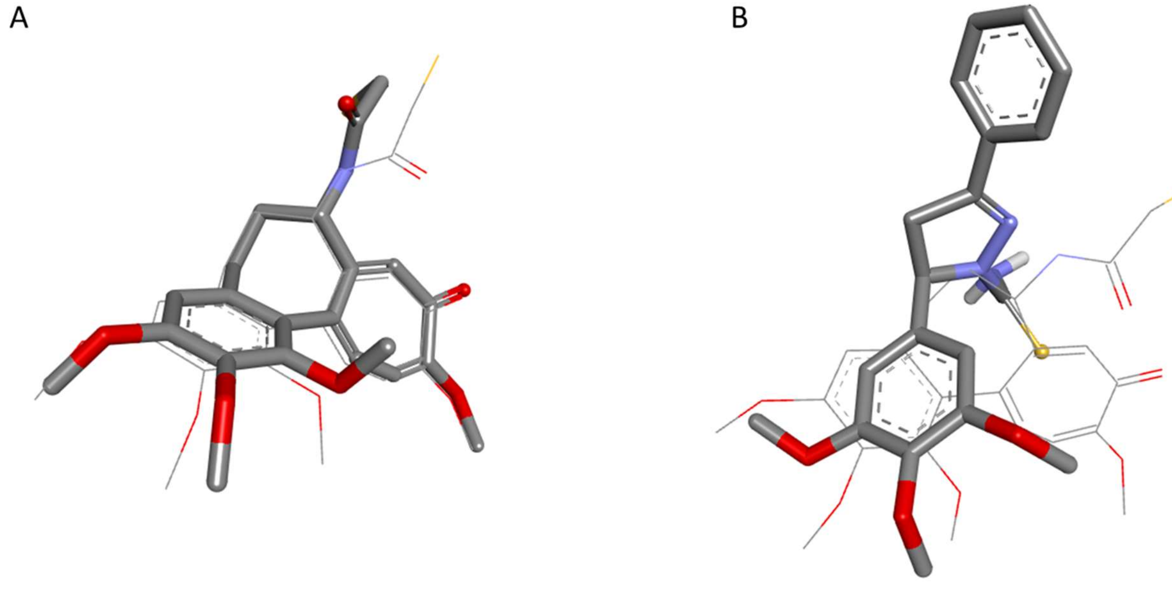

Supplementary Figure 4: *A.* Docking of colchicine (thick lines) into tubulin and overlap with the colchicine (thin lines) from the x-ray crystal structure. *B*. Docking results of the SSE15206 (thick lines) and its overlap with the colchicine (thin lines) from the crystal structure.

Experimental section

Synthesis and characterization of the in-house compound library:

All commercial reagents were used without further purification. All solvents were of reagent grade and used after distillation. Column chromatography was carried out on silica gel 60 (230–400 mesh) from Merck. Yields refer to chromatographically and spectroscopically pure compounds unless otherwise stated. Infrared spectra were recorded on Bruker Alpha spectrophotometer. Proton and carbon NMR spectra were recorded on a 500 MHz spectrometer. High resolution mass spectra were recorded on Thermo LTQ Velos Orbitrap mass spectrometer.

Typical method for the synthesis of Chalcones 1-16: In a typical experiment, appropriate aldehyde (1 mmol) was added to Ethanol (10 mL) in a round bottom flask (25 mL). Then appropriate acetophenone (1 mmol) was added to the reaction mixture followed by addition of 15 N ethanolic solution of sodium ethoxide (0.2 mL). The reaction mixture was stirred overnight (15-17 hours) at room temperature. Then the solvent was removed and the mixture was dissolved in ethyl acetate. The contents of the flask were then transferred to the separating funnel and washed with brine. Then the organic layer was dried over $MgSO_4$ and the solvent was removed to obtain the product. In case the product was oil, it was purified by column chromatography in EtOAc:hexane and in case of the solid, it was purified by recrystallization from ethanol.

(E)-chalcone (1): 74% ^{1}H NMR (500 MHz, CHLOROFORM-*d*) δ ppm 7.44 (m, 3 H), 7.54 (m, 3 H), 7.61 (t, *J*=7 Hz, 1 H), 7.67 (m, 2 H), 7.83 (d, *J*=16 Hz, 1 H), 8.04 (d, *J*=7 Hz, 2 H) ^{13}C NMR (126 MHz, CHLOROFORM-*d*) δ ppm 122.0 (CH), 128.4 (CH), 128.5 (CH), 128.6 (CH), 128.9 (CH), 130.5 (CH), 132.8 (CH), 134.8, 138.1, 144.8 (CH), 190.5 IR (cm^{-1}) neat 1660,1601 MS (APCI) m/z: 209.1 [M+H]$^+$ m.p. 60 ˚C HRMS calculated for $C_{15}H_{13}O$: 209.09609 [M+H]$^+$ found 209.09548

(E)-3-(3-nitrophenyl)-1-phenylprop-2-en-1-one (2): 89% ^{1}H NMR (500 MHz, CHLOROFORM-*d*) δ ppm 7.55 (t, *J*=7.7 Hz, 2 H), 7.63 (m, 2 H), 7.67 (d, *J*=15.8 Hz, 1 H), 7.85 (d, *J*=15.7 Hz, 1 H), 7.94 (d, *J*=7.6 Hz, 1 H), 8.06 (d, *J*=7.4 Hz, 2 H), 8.27 (d, *J*=7.4 Hz, 1 H), 8.53 (s, 1 H) ^{13}C NMR (126 MHz, CHLOROFORM-*d*) δ ppm 122.3 (CH), 124.5 (CH), 124.7 (CH), 128.6 (CH), 128.8 (CH), 130.0 (CH), 133.3 (CH), 134.4 (CH), 136.59, 137.51, 141.6 (CH), 148.66, 189.63 IR (cm^{-1}) neat 1660,1594 MS (APCI) m/z: 254.1 [M+H]$^+$ m.p. 150-152 ˚C HRMS calculated for $C_{15}H_{12}O_3N$: 254.08117 [M+H]$^+$ found: 254.08020

(E)-3-(4-methoxyphenyl)-1-phenylprop-2-en-1-one (3): 88% ^{1}H NMR (500 MHz, CHLOROFORM-*d*) δ ppm 3.86 (m, 3 H), 6.95 (d, *J*=8.7 Hz, 2 H), 7.43 (d, *J*=15.7 Hz, 1 H), 7.51 (t, *J*=7.6 Hz, 2 H), 7.59 (t, *J*=7.2 Hz, 1 H), 7.62 (d, *J*=8.7 Hz, 2 H), 7.80 (d, *J*=15.5 Hz, 1 H), 8.02 (d, *J*=7.7 Hz, 2 H) ^{13}C NMR (126 MHz, CHLOROFORM-*d*) δ ppm 55.4 (CH_3), 114.4 (CH), 119.7 (CH), 127.5, 128.4 (CH), 128.5 (CH), 130.2 (CH), 132.5 (CH), 138.4, 144.7 (CH), 161.6, 190.6 IR (cm^{-1}) neat 1656,1594 MS (APCI) m/z: 239.1 [M+H]$^+$ m.p. 79-80 ˚C HRMS calculated for $C_{16}H_{15}O_2$: 239.10666 [M+H]$^+$ found: 239.10555

(E)-3-(4-fluorophenyl)-1-phenylprop-2-en-1-one (4): 83% ^{1}H NMR (500 MHz, CHLOROFORM-*d*) δ ppm 7.13 (t, *J*=8.6 Hz, 2 H), 7.48 (d, *J*=15.5 Hz, 1 H), 7.52 (t, *J*=7.7 Hz, 2 H), 7.61 (t, *J*=7.4 Hz, 1

H), 7.65 (dd, *J*=8.5, 5.6 Hz, 2 H), 7.79 (d, *J*=15.7 Hz, 1 H), 8.03 (d, *J*=7.7 Hz, 2 H) ^{13}C NMR (126 MHz, CHLOROFORM-*d*) δ ppm 116.13 (d, *J*=21.8 Hz, CH), 121.69 (d, *J*=2.5 Hz, CH), 128.46 (CH), 128.64 (CH), 130.34 (d, *J*=8.7 Hz, CH), 131.09 (d, *J*=3.3 Hz), 132.86 (CH), 138.07, 143.51(CH), 164.03 (d, *J*=253.4 Hz), 190.31 IR (cm^{-1}) neat 1658,1587 MS (APCI) m/z: 227.1 $[M+H]^+$ m.p. 91 ˚C HRMS calculated for $C_{15}H_{12}OF$: 227.08667 $[M+H]^+$ found: 227.08586

(E)-1-(4-methoxyphenyl)-3-phenylprop-2-en-1-one (5): 61% ^{1}H NMR (500 MHz, CHLOROFORM-*d*) δ ppm 3.90 (s, 3 H), 7.00 (d, *J*=8.8 Hz, 2 H), 7.43 (m, 3 H), 7.57 (d, *J*=15.7 Hz, 1 H), 7.66 (m, 2 H), 7.82 (d, *J*=15.7 Hz, 1 H), 8.06 (d, *J*=8.8 Hz, 2 H) ^{13}C NMR (126 MHz, CHLOROFORM-*d*) δ ppm 55.5 (CH_3), 113.8 (CH), 121.8 (CH), 128.3 (CH), 128.9 (CH), 130.3 (CH), 130.8 (CH), 131.0, 135.0, 143.9 (CH), 163.4, 188.7 IR (cm^{-1}) neat 1652,1597 MS (APCI) m/z: 239.1 $[M+H]^+$ m.p. 114 ˚C HRMS calculated for $C_{16}H_{15}O_2$: 239.10666 $[M+H]^+$ found: 239.10568

(E)-1-phenyl-3-(3,4,5-trimethoxyphenyl)prop-2-en-1-one (6): 91% ^{1}H NMR (500 MHz, CHLOROFORM-*d*) δ ppm 3.91 (s, 3 H), 3.93 (s, 6 H), 6.87 (s, 2 H), 7.42 (d, *J*=15.7 Hz, 1 H), 7.52 (t, *J*=7.6 Hz, 2 H), 7.59 (t, *J*=7.3 Hz, 1 H), 7.73 (d, *J*=15.7 Hz, 1 H), 8.02 (d, *J*=7.7 Hz, 2 H) ^{13}C NMR (126 MHz, CHLOROFORM-*d*) δ ppm 56.6 (CH_3), 61.4 (CH_3), 105.9 (CH), 121.8 (CH), 128.9 (CH), 129.0 (CH), 130.7, 133.1 (CH), 138.6, 140.7, 145.4 (CH), 153.8, 191.0 IR (cm^{-1}) neat 1659, 1575 MS (APCI) m/z: 299.1 $[M+H]^+$ m.p. 104 ˚C HRMS calculated for $C_{18}H_{19}O_4$: 299.12779 $[M+H]^+$ found: 299.12674

(E)-3-(4-(benzyloxy)phenyl)-1-phenylprop-2-en-1-one (7): 85% ^{1}H NMR (500 MHz, CHLOROFORM-*d*) δ ppm 5.13 (s, 2 H), 7.03 (d, *J*=8.7 Hz, 2 H), 7.36 (t, *J*=6.7 Hz, 1 H), 7.43 (m, 5 H), 7.51 (t, *J*=7.7 Hz, 2 H), 7.60 (m, 3 H), 7.80 (d, *J*=15.7 Hz, 1 H), 8.03 (d, *J*=7.6 Hz, 2 H) ^{13}C NMR (126 MHz, CHLOROFORM-*d*) δ ppm 70.1 (CH_2), 115.2 (CH), 119.8 (CH), 127.5 (CH), 127.8, 128.2 (CH), 128.4 (CH), 128.5 (CH), 128.7 (CH), 130.2 (CH), 132.6 (CH), 136.3, 138.4, 144.6 (CH), 160.8, 190.6 IR (cm^{-1}) neat 1651,1587 MS (APCI) m/z: 315.1 $[M+H]^+$ m.p. 120 ˚C HRMS calculated for $C_{22}H_{19}O_2$: 315.13796 $[M+H]^+$ found: 315.13682

(E)-1-(4-(benzyloxy)phenyl)-3-phenylprop-2-en-1-one (8): 95% ^{1}H NMR (500 MHz, CHLOROFORM-*d*) δ ppm 5.17 (s, 2 H), 7.08 (d, *J*=8.8 Hz, 2 H), 7.37 (t, *J*=6.9 Hz, 1 H), 7.44 (m, 7 H), 7.57 (d, *J*=15.7 Hz, 1 H), 7.66 (m, 2 H), 7.82 (d, *J*=15.7 Hz, 1 H), 8.06 (d, *J*=8.8 Hz, 2 H) ^{13}C NMR (126 MHz, CHLOROFORM-*d*) δ ppm 70.1 (CH_2), 114.7 (CH), 121.8 (CH), 127.5 (CH), 128.2 (CH), 128.3 (CH), 128.7 (CH), 128.9 (CH), 130.3 (CH), 130.8 (CH), 131.2, 135.0, 136.1, 144.0 (CH), 162.5, 188.7 IR (cm^{-1}) neat 1655, 1595 MS (APCI) m/z: 315.1 $[M+H]^+$ m.p. 122 ˚C HRMS calculated for $C_{22}H_{19}O_2$: 315.13796 $[M+H]^+$ found: 315.13671

(E)-1-(4-(benzyloxy)phenyl)-3-(4-chlorophenyl)prop-2-en-1-one (9): 68% ^{1}H NMR (500 MHz, CHLOROFORM-*d*) δ ppm 5.17 (s, 2 H), 7.08 (d, *J*=8.9 Hz, 2 H), 7.40 (m, 7 H), 7.53 (d, *J*=15.7 Hz, 1 H), 7.58 (d, *J*=8.5 Hz, 2 H), 7.76 (d, *J*=15.5 Hz, 1 H), 8.05 (d, *J*=8.5 Hz, 2 H) ^{13}C NMR (126 MHz, CHLOROFORM-*d*) δ ppm 70.1 (CH_2), 114.7 (CH), 122.2 (CH), 127.5 (CH), 128.3 (CH), 128.7 (CH), 129.2 (CH), 129.5 (CH), 130.8 (CH), 131.0, 133.5, 136.1, 136.2, 142.5 (CH), 162.6, 188.3 IR (cm^{-1}) neat 1652,1589 MS (APCI) m/z: 349.1 $[M+H]^+$ m.p. 176 ˚C HRMS calculated for $C_{22}H_{18}O_2Cl$: 349.09898 $[M+H]^+$ found: 349.09791

(E)-1-(4-(benzyloxy)phenyl)-3-(3-methoxyphenyl)prop-2-en-1-one (10): 76% ^{1}H NMR (500 MHz, CHLOROFORM-*d*) δ ppm 3.87 (s, 3 H), 5.17 (s, 2 H), 6.97 (dd, *J*=8.1, 2.2 Hz, 1 H), 7.07 (d, *J*=8.7 Hz, 2 H), 7.17 (s, 1 H), 7.26 (d, *J*=7.8 Hz, 1 H), 7.36 (m, 2 H), 7.42 (t, *J*=7.0 Hz, 2 H), 7.46 (d, *J*=7.0 Hz, 2 H), 7.54 (d, *J*=15.7 Hz, 1 H), 7.78 (d, *J*=15.5 Hz, 1 H), 8.05 (d, *J*=8.8 Hz, 2 H) ^{13}C NMR (126 MHz, CHLOROFORM-*d*) δ ppm 55.3 (CH_3), 70.1 (CH_2), 113.3 (CH), 114.7 (CH), 116.1 (CH), 121.0 (CH), 122.1 (CH), 127.5 (CH), 128.2 (CH), 128.7 (CH), 129.9 (CH), 130.8 (CH), 131.2, 136.1, 136.4, 143.9 (CH), 159.9, 162.5, 188.7 IR (cm^{-1}) neat 1655,1597 MS (APCI) m/z: 345.1 $[M+H]^+$ m.p. 157 ˚C HRMS calculated for $C_{23}H_{21}O$: 345.14852 $[M+H]^+$ found: 345.14707

(E)-1-(4-(benzyloxy)phenyl)-3-(4-methoxyphenyl)prop-2-en-1-one (11): 67% ^{1}H NMR (500 MHz, CHLOROFORM-*d*) δ ppm 3.87 (s, 3 H), 5.16 (s, 2 H), 6.95 (d, *J*=8.7 Hz, 2 H), 7.07 (d, *J*=8.8 Hz, 2 H), 7.36 (t, *J*=7.3 Hz, 1 H), 7.44 (m, 5 H), 7.61 (d, *J*=8.5 Hz, 2 H), 7.80 (d, *J*=15.5 Hz, 1 H), 8.05 (d, *J*=8.8 Hz, 2 H) ^{13}C NMR (126 MHz, CHLOROFORM-*d*) δ ppm 55.4 (CH_3), 70.1 (CH_2), 114.3 (CH), 114.6 (CH), 119.4 (CH), 127.5 (CH), 127.7, 128.2 (CH), 128.7 (CH), 130.1 (CH), 130.7 (CH), 131.5, 136.2, 143.8 (CH), 161.5, 162.4, 188.7 IR (cm^{-1}) neat 1653,1594 MS (APCI) m/z: 345.1 $[M+H]^+$ m.p. 140 ˚C HRMS calculated for $C_{23}H_{21}O_3$: 345.14852 $[M+H]^+$ found: 345.14740

(E)-1-(4-(benzyloxy)phenyl)-3-(4-fluorophenyl)prop-2-en-1-one (12): 92% ^{1}H NMR (500 MHz, CHLOROFORM-*d*) δ ppm 5.17 (s, 2 H), 7.07 (d, *J*=8.8 Hz, 2 H), 7.12 (t, *J*=8.6 Hz, 2 H), 7.37 (t, *J*=6.9 Hz, 1 H), 7.45 (m, 5 H), 7.64 (dd, *J*=8.5, 5.4 Hz, 2 H), 7.78 (d, *J*=15.7 Hz, 1 H), 8.05 (d, *J*=8.8 Hz, 2 H) ^{13}C NMR (126 MHz, CHLOROFORM-*d*) δ ppm 70.1 (CH_2), 114.7 (CH), 116.1 (d, *J*=22.3 Hz, CH), 121.5 (d, *J*=2.2 Hz, CH), 127.5 (CH), 128.3 (CH), 128.7 (CH), 130.2 (d, *J*=8.4 Hz, CH), 130.8 (CH), 131.1, 131.3 (d, *J*=3.5 Hz), 136.1, 142.7 (CH), 162.6, 163.9 (d, *J*=251.2 Hz, 1 C), 188.4 IR (cm^{-1}) neat 1656,1596 MS (APCI) m/z: 333.1 $[M+H]^+$ m.p. 116 ˚C HRMS calculated for $C_{22}H_{18}O_2F$: 333.12853 $[M+H]^+$ found: 333.12734

(E)-1-(4-(benzyloxy)phenyl)-3-(2-chlorophenyl)prop-2-en-1-one (13): 98% ^{1}H NMR (500 MHz, CHLOROFORM-*d*) δ ppm 5.17 (s, 2 H), 7.08 (d, *J*=8.9 Hz, 2 H), 7.33 (m, 2 H), 7.36 (t, *J*=7.0 Hz, 1 H), 7.42 (t, *J*=7.4 Hz, 2 H), 7.45 (m, 3 H), 7.51 (d, *J*=15.8 Hz, 1 H), 7.76 (m, 1 H), 8.05 (d, *J*=8.8 Hz, 2 H), 8.18 (d, *J*=15.8 Hz, 1 H) ^{13}C NMR (126 MHz, CHLOROFORM-*d*) δ ppm 70.1 (CH_2), 114.7 (CH), 124.6 (CH), 127.0 (CH), 127.4, 127.5 (CH), 127.7 (CH), 128.2 (CH), 128.7 (CH), 130.2 (CH), 130.9 (CH), 131.0 (CH), 133.4, 135.4, 136.1, 139.8 (CH), 162.6, 188.6 IR (cm^{-1}) neat 1652, 1595 MS (APCI) m/z: 349.1 $[M+H]^+$ m.p. 125 ˚C HRMS calculated for $C_{22}H_{18}O_2Cl$: 349.09898 $[M+H]^+$ found: 349.09808

(E)-1-(4-((4-methylbenzyl)oxy)phenyl)-3-phenylprop-2-en-1-one (14): 93% ^{1}H NMR (500 MHz, CHLOROFORM-*d*) δ ppm 2.39 (s, 3 H), 5.12 (s, 2 H), 7.07 (d, *J*=8.8 Hz, 2 H), 7.23 (d, *J*=7.7 Hz, 2 H), 7.35 (d, *J*=7.8 Hz, 2 H), 7.43 (m, 3 H), 7.56 (d, *J*=15.7 Hz, 1 H), 7.66 (m, 2 H), 7.82 (d, *J*=15.7 Hz, 1 H), 8.06 (d, *J*=8.8 Hz, 2 H) ^{13}C NMR (126 MHz, CHLOROFORM-*d*) δ ppm 21.2 (CH_3), 70.1 (CH_2), 114.7 (CH), 121.8 (CH), 127.6 (CH), 128.3 (CH), 128.9 (CH), 129.4 (CH), 130.3 (CH), 130.8 (CH), 131.1, 133.1, 135.0, 138.1, 143.9 (CH), 162.6, 188.7 IR (cm^{-1}) neat 1656, 1574 MS (APCI) m/z: 329.2 $[M+H]^+$ m.p. 145 ˚C HRMS calculated for $C_{23}H_{21}O_2$: 329.15361 $[M+H]^+$ found: 329.15246

(E)-3-(4-fluorophenyl)-1-(4-((4-methylbenzyl)oxy)phenyl)prop-2-en-1-one (15): 83% ^{1}H NMR (500 MHz, CHLOROFORM-*d*) δ ppm 2.38 (s, 2 H), 5.12 (s, 2 H), 7.06 (d, *J*=8.7 Hz, 2 H), 7.12 (t, *J*=8.5 Hz, 2 H), 7.23 (d, *J*=7.7 Hz, 2 H), 7.34 (d, *J*=7.8 Hz, 2 H), 7.48 (d, *J*=15.7 Hz, 1 H), 7.64 (dd, *J*=8.1, 5.6 Hz, 2 H), 7.78 (d, *J*=15.7 Hz, 1 H), 8.04 (d, *J*=8.7 Hz, 2 H) ^{13}C NMR (126 MHz, CHLOROFORM-*d*) δ

ppm 21.2 (CH_3), 70.1 (CH_2), 114.7 (CH), 116.0 (d, *J*=22.1 Hz, CH), 121.5 (d, *J*=2.2 Hz, CH), 127.6 (CH), 129.4 (CH), 130.2 (d, *J*=8.7 Hz, CH), 130.8 (CH), 131.0, 131.3 (d, *J*=3.5 Hz), 133.0, 138.1, 142.6 (CH), 162.7, 163.9 (d, *J*=251.2 Hz), 188.4 IR (cm^{-1}) neat 1656, 1594 MS (APCI) m/z: 347.1 $[M+H]^+$ m.p. 166 ˚C HRMS calculated for $C_{23}H_{20}O_2F$: 347.14418 $[M+H]^+$ found: 347.14283

(E)-3-(4-chlorophenyl)-1-(4-((4-methylbenzyl)oxy)phenyl)prop-2-en-1-one (16): 69% ^{1}H NMR (500 MHz, CHLOROFORM-*d*) δ ppm 2.38 (s, 3 H), 5.12 (s, 2 H), 7.06 (d, *J*=8.4 Hz, 2 H), 7.23 (d, *J*=7.7 Hz, 2 H), 7.34 (d, *J*=7.8 Hz, 2 H), 7.40 (d, *J*=8.4 Hz, 2 H), 7.53 (d, *J*=15.7 Hz, 1 H), 7.58 (d, *J*=8.4 Hz, 2 H), 7.76 (d, *J*=15.5 Hz, 1 H), 8.04 (d, *J*=8.7 Hz, 2 H) ^{13}C NMR (126 MHz, CHLOROFORM-*d*) δ ppm 21.2 (CH_3), 70.1 (CH_2), 114.7 (CH), 122.2 (CH), 127.7 (CH), 129.2 (CH), 129.4 (CH), 129.5 (CH), 130.8 (CH), 131.0, 133.0, 133.5, 136.1, 138.1, 142.4 (CH), 162.7, 188.3 IR (cm^{-1}) neat 1663,1626,1599 MS (APCI) m/z: 363.1 $[M+H]^+$ m.p. 185 ˚C HRMS calculated for $C_{23}H_{20}O_2Cl$: 363.11463 $[M+H]^+$ found: 363.11353

Typical method for the synthesis of SSE15201-SSE15216: In a typical procedure, the chalcone (1 mmol) was dissolved in 10 mL of a mixture of NaOH in ethanol. Then thiosemicarbazide (1 mmol) was added to the reaction mixture and the mixture was reflux with for 5 hours. Then the reaction mixture was cooled resulting in the precipitation of the desired product precipitated. The precipitates were filtered under vacuum and dried. Recrystallized was performed using ethanol as solvent.

3,5-diphenyl-4,5-dihydro-1H-pyrazole-1-carbothioamide (SSE15201): 55% ^{1}H NMR (700 MHz, CHLOROFORM-*d*) δ ppm 7.75 (d, *J*=7.3 Hz, 2 H) 7.48 (t, *J*=7.5 Hz, 1 H) 7.45 (t, *J*=7.4 Hz, 2 H) 7.35 (t, *J*=7.4 Hz, 2 H) 7.28 (d, *J*=7.5 Hz, 1 H) 7.25 (d, *J*=7.5 Hz, 2 H) 6.07 (dd, *J*=11.6, 3.7 Hz, 1 H) 3.87 (dd, *J*=17.6, 11.4 Hz, 1 H) 3.2 (dd, *J*=17.6, 3.7 Hz, 1 H) ^{13}C NMR (176 MHz, CHLOROFORM-*d*) δ ppm 176.6, 156.1, 141.7, 131.0 (CH), 130.5, 128.9 (CH), 128.8 (CH), 127.6 (CH), 127.0 (CH), 125.3 (CH), 63.4 (CH), 43.1 (CH_2) IR (cm^{-1}) neat 3413, 3261 MS (APCI) m/z: 282.1 $[M+H]^+$ m.p. 186-187 ˚C HRMS calculated for $C_{16}H_{16}N_3S^+$: 282.1059 $[M+H]^+$ found 282.1059

5-(3-nitrophenyl)-3-phenyl-4,5-dihydro-1H-pyrazole-1-carbothioamide (SSE15202): 84% ^{1}H NMR (700 MHz, CHLOROFORM-*d*) δ ppm 8.14 (d, *J*=8.17 Hz, 1 H) 8.10 (s, 1 H) 7.75 (d, *J*=7.31 Hz, 2 H) 7.60 (d, *J*=7.74 Hz, 1 H) 7.53 (t, *J*=7.74 Hz, 1 H) 7.50 (t, *J*=7.31 Hz , 1 H) 7.46 (t, *J*=7.74 Hzm 2 H) 6.15 (dd, *J*=11.62, 4.30 Hz, 1 H) 3.95 (dd, *J*=17.85, 11.83 Hz, 1 H) 3.23 (dd, *J*=17.64, 4.30 Hz, 1 H) ^{13}C NMR (176 MHz, CHLOROFORM-*d*) δ 176.8, 155.6, 148.6, 143.8, 131.9 (CH), 131.4 (CH), 130.0, 129.9 (CH), 129.0 (CH), 127.0 (CH), 122.7 (CH), 120.7 (CH), 62.7 (CH), 42.8 (CH_2) IR (cm^{-1}) neat 3403, 3229 MS (APCI) m/z: 327.1 $[M+H]^+$ m.p. 175-176 ˚C HRMS calculated for $C_{16}H_{15}N_4O_2S^+$: 327.0910 $[M+H]^+$ found 327.0910

5-(4-methoxyphenyl)-3-phenyl-4,5-dihydro-1H-pyrazole-1-carbothioamide (SSE15203): 69% ^{1}H NMR (700 MHz, CHLOROFORM-d) δ 7.72 - 7.77 (d, *J* = 7.10 Hz, 2H), 7.42 - 7.49 (m, 3H), 7.17 (d, *J* = 8.60 Hz, 2H), 6.86 (d, *J* = 8.60 Hz, 2H), 6.00 (dd, *J* = 3.44, 11.40 Hz, 1H), 3.83 (dd, *J* = 11.40, 17.64 Hz, 1H), 3.78 (s, 3H), 3.22 (dd, *J* = 3.44, 17.64 Hz, 1H) ^{13}C NMR (176 MHz, CHLOROFORM-d) δ 176.6, 158.9, 156.0, 133.9, 131.0 (CH), 130.6, 128.8 (CH), 126.9 (CH), 126.7 (CH), 114.2 (CH), 62.9(CH), 55.2(CH_3), 43.1 (CH_2) IR (cm^{-1}) neat 3384, 3255 MS (APCI) m/z: 312.1 $[M+H]^+$ m.p. 176-178 ˚C HRMS calculated for $C_{17}H_{18}N_3OS^+$: 312.1165 $[M+H]^+$ found 312.1165

5-(4-fluorophenyl)-3-phenyl-4,5-dihydro-1H-pyrazole-1-carbothioamide (SSE15204): 94% ^{1}H NMR (700 MHz, CHLOROFORM-d) δ 7.74 (d, *J* = 7.53 Hz, 2H), 7.49 (t, *J* = 7.31 Hz,, 1H), 7.45 (t, *J* = 7.31 Hz, 2H), 7.22 (dd, *J* = 5.38, 8.39 Hz, 2H), 7.02 (t, *J* = 8.60 Hz, 2H), 6.03 (dd, *J* = 3.55, 11.51 Hz, 1H), 3.86 (dd, *J* = 11.40, 17.64 Hz, 1H), 3.21 (dd, *J* = 3.66, 17.64 Hz, 1H) ^{13}C NMR (176 MHz, CHLOROFORM-d) δ 176.7, 162.1 (d, *J*=245 Hz), 155.9, 137.6 (d, *J*=3.81 Hz, CH) , 131.1 (CH), 130.4, 128.9 (CH), 127.3(d, *J*=7.6 Hz, CH), 126.9 (CH), 115.8 (d, *J*=21.62 Hz, CH), 62.8(CH), 43.0(CH_2) IR (cm^{-1}) neat 3471, 3352 MS (APCI) m/z: 300.1 $[M+H]^+$ m.p. 249-250 ˚C HRMS calculated for $C_{16}H_{15}FN_3S^+$: 300.0965 $[M+H]^+$ found 300.0965

3-(4-methoxyphenyl)-5-phenyl-4,5-dihydro-1H-pyrazole-1-carbothioamide (SSE15205): 75% ^{1}H NMR (700 MHz, CHLOROFORM-*d*) δ 7.68 (d, *J* = 8.60 Hz, 2H), 7.34 (t, *J* = 7.74 Hz, 2H), 7.26 - 7.29 (t, *J* = 7.31 Hz, 1H), 7.23 (d, *J* = 7.74 Hz, 2H), 6.94 (d, *J* = 8.60 Hz, 2H), 6.03 (dd, *J* = 3.01, 11.19 Hz, 2H), 3.86 (s, 3H), 3.82 (dd, *J* = 11.62, 17.64 Hz, 1H), 3.19 (dd, *J* = 3.23, 17.42 Hz, 1H) ^{13}C NMR (176 MHz, CHLOROFORM-*d*) δ 176.2, 161.9, 155.8, 141.8, 128.9 (CH), 128.6 (CH), 127.6 (CH), 125.4 (CH), 123.1, 114.2 (CH), 63.3 (CH), 55.4 (CH_3), 43.1 (CH_2) IR (cm^{-1}) neat 3481, 3343 MS (APCI) m/z: 312.1 $[M+H]^+$ m.p. 182-183 ˚C HRMS calculated for $C_{17}H_{18}N_3OS^+$: 312.1165 $[M+H]^+$ found 312.1165

3-phenyl-5-(3,4,5-trimethoxyphenyl)-4,5-dihydro-1H-pyrazole-1-carbothioamide (SSE15206): 93% ^{1}H NMR (700 MHz, CHLOROFORM-d) δ 7.73 - 7.76 (m, 2H), 7.46 - 7.49 (m, 1H), 7.42 - 7.46 (m, 2H), 6.42 (s, 2H), 5.99 (dd, *J* = 3.66, 11.40 Hz, 1H), 3.80 - 3.88 (m, 10H), 3.23 (dd, *J* = 3.66, 17.64 Hz, 1H) ^{13}C NMR (176 MHz, CHLOROFORM-d) δ 176.8, 137.5, 137.1, 131.1(CH), 130.5, 128.9(CH), 126.9(CH), 102.0(CH), 77.2, 76.8, 63.6 (CH), 60.8(CH_3), 56.1(CH_3), 43.3 (CH_2) IR (cm^{-1}) neat 3468, 3337 MS (APCI) m/z: 372.1 $[M+H]^+$ m.p. 164-165 ˚C HRMS calculated for $C_{19}H_{22}N_3O_3S^+$: 372.1376 $[M+H]^+$ found 372.1376

5-(4-(benzyloxy)phenyl)-3-phenyl-4,5-dihydro-1H-pyrazole-1-carbothioamide (SSE15207): 94% ^{1}H NMR (700 MHz, CHLOROFORM-*d*) δ ppm 7.74 (d, *J*=7.31 Hz, 2 H) 7.41 - 7.49 (m, 5H) 7.38 (t, *J*=7.53 Hz, 2H) 7.33 (t, *J*=7.31 Hz, 1H) 7.18 (d, *J*=8.39 Hz, 2H) 6.94 (d, *J*=8.39 Hz, 2H) 6.01 (dd, *J*=11.40, 3.23 Hz, 1H) 5.03 (s, 2H) 3.83 (dd, *J*=17.64, 11.40 Hz, 1H) 3.22 (dd, *J*=17.64, 3.23 Hz, 1H) ^{13}C NMR (176 MHz, CHLOROFORM-*d*) δ ppm 176.6 158.2, 156.0, 136.9, 134.2, 131.0, 130.6, 128.8 (CH), 128.6 (CH), 128.0 (CH), 127.5 (CH), 126.9 (CH), 126.8 (CH), 115.0 (CH), 70.0 (CH_2), 62.93(CH), 43.1 (CH_2) IR (cm^{-1}) neat 3437, 3279 MS (APCI) m/z: 388.1 $[M+H]^+$ m.p. 201-202 ˚C HRMS calculated for $C_{23}H_{22}N_3OS^+$: 388.1478 $[M+H]^+$ found 388.1478

3-(4-(benzyloxy)phenyl)-5-phenyl-4,5-dihydro-1H-pyrazole-1-carbothioamide (SSE15208): 63% ^{1}H NMR (700 MHz, CHLOROFORM-d) δ 7.67 (d, *J* = 7.74 Hz, 2H), 7.38 - 7.47 (m, 4H), 7.31 - 7.37 (m, 3H), 7.26 (s, 1H), 7.23 (d, *J* = 7.10 Hz, 2H), 7.01 (d, *J* = 7.96 Hz, 2H), 6.03 (d, *J* = 11.19 Hz, 2H), 3.81 (dd, *J* = 12.05, 16.78 Hz, 1H), 3.18 (d, *J* = 17.64 Hz, 1H) ^{13}C NMR (176 MHz, CHLOROFORM-d) δ 176.2, 161.0, 155.8, 141.8, 136.2, 128.9(CH), 128.7(CH), 128.6(CH), 128.2(CH), 127.6(CH), 127.4(CH), 125.3(CH), 123.3, 115.1(CH), 70.1(CH_2), 63.3, 43.1(CH_2) IR (cm^{-1}) neat 3388, 3248 MS (APCI) m/z: 388.1 $[M+H]^+$ m.p. 189-191 ˚C HRMS calculated for $C_{23}H_{22}N_3OS^+$: 388.1478 $[M+H]^+$ found 388.1478

3-(4-(benzyloxy)phenyl)-5-(4-chlorophenyl)-4,5-dihydro-1H-pyrazole-1-carbothioamide (SSE15209): 75% ^{1}H NMR (700 MHz, CHLOROFORM-d) δ 7.67 (d, *J* = 7.74 Hz, 2H), 7.38 - 7.46 (m,

4H), 7.37-7.35 (m, 1H), 7.30 (d, *J* = 7.53 Hz, 2H), 7.17 (d, *J* = 7.53 Hz, 2H), 7.02 (d, *J* = 7.74 Hz, 2H), 5.99 (d, *J* = 11.40 Hz, 1H), 5.13 (s, 2H), 3.82 (dd, *J* = 11.94, 17.10 Hz, 1H), 3.14 (d, *J* = 17.42 Hz, 1H) ^{13}C NMR (176 MHz, CHLOROFORM-d) δ 176.2, 161.1, 155.7, 140.3, 136.1, 133.3, 129.0(CH), 128.8(CH), 128.7(CH), 128.3(CH), 127.4(CH), 126.9(CH), 123.1, 115.2, 70.1(CH_2), 62.7(CH), 43.0(CH_2) IR (cm^{-1}) neat 3435, 3359 MS (APCI) m/z: 422.1 $[M+H]^+$ m.p. 168-169 ˚C HRMS calculated for $C_{23}H_{21}ClN_3OS^+$: 422.1088 $[M+H]^+$ found 422.1088

3-(4-(benzyloxy)phenyl)-5-(3-methoxyphenyl)-4,5-dihydro-1H-pyrazole-1-carbothioamide (SSE15210): 72% ^{1}H NMR (700 MHz, CHLOROFORM-d) δ 7.67 (d, *J* = 8.82 Hz, 2H), 7.44 (d, *J* = 7.31 Hz, 2H), 7.41 (t, *J* = 7.31 Hz, 2H), 7.35 (t, *J* = 7.31 Hz, 1H), 7.25 (d, *J* = 7.96 Hz, 1H), 7.01 (d, *J* = 9.03 Hz, 2H), 6.78 - 6.83 (m, 2H), 6.76 (d, *J* = 1.72 Hz, 1H), 6.00 (dd, *J* = 3.44, 11.40 Hz, 1H), 5.12 (s, 2H), 3.82-3.78 (m, 4H), 3.17 (dd, *J* = 3.55, 17.53 Hz, 1H) ^{13}C NMR (176 MHz, CHLOROFORM-d) δ 176.2, 161.0, 159.9, 155.8, 143.4, 136.2, 130.0(CH), 128.7(CH), 128.6(CH), 128.2(CH), 127.4(CH), 123.3, 117.5(CH), 115.1(CH), 112.5(CH), 111.4(CH), 70.1(CH_2), 63.2(CH), 55.2(CH_3), 43.1(CH_2) IR (cm^{-1}) neat 3402, 3241 MS (APCI) m/z: 418.2 $[M+H]^+$ m.p. 170-171 ˚C HRMS calculated for $C_{24}H_{24}N_3O_2S^+$: 418.1584 $[M+H]^+$ found 418.1584

3-(4-(benzyloxy)phenyl)-5-(4-methoxyphenyl)-4,5-dihydro-1H-pyrazole-1-carbothioamide (SSE15211): 63% ^{1}H NMR (700 MHz, CHLOROFORM-d) δ 7.68 (d, *J* = 8.60 Hz, 2H), 7.44 (d, *J* = 7.53 Hz, 2H), 7.41 (t, *J* = 7.53 Hz, 2H), 7.36 (t, *J* = 7.31 Hz 1H), 7.16 (d, *J* = 8.60 Hz, 2H), 7.02 (d, *J* = 8.82 Hz, 2H), 6.86 (d, *J* = 8.60 Hz, 2H), 5.98 (dd, *J* = 3.33, 11.29 Hz, 1H), 5.13 (s, 7H), 3.78 (m, 4H), 3.17 (dd, *J* = 3.44, 17.64 Hz, 1H) ^{13}C NMR (176 MHz, CHLOROFORM-d) δ 176.1, 161.0, 158.9, 155.9, 136.2, 134.0, 128.7(CH), 128.6(CH), 128.2(CH), 127.4(CH), 126.8(CH), 123.4, 115.1(CH), 114.1(CH), 70.1(CH_2), 62.8(CH), 55.2(CH_3), 43.1(CH_2) IR (cm^{-1}) neat 3447, 3365 MS (APCI) m/z: 418.2 $[M+H]^+$ m.p. 128-129 ˚C HRMS calculated for $C_{24}H_{24}N_3O_2S^+$: 418.1584 $[M+H]^+$ found 418.15837

3-(4-(benzyloxy)phenyl)-5-(4-fluorophenyl)-4,5-dihydro-1H-pyrazole-1-carbothioamide (SSE15212): 85% ^{1}H NMR (700 MHz, CHLOROFORM-*d*) δ 7.68 (d, *J* = 8.60 Hz, 2H), 7.44 (d, *J* = 7.31 Hz, 2H), 7.41 (t, *J* = 7.31 Hz, 2H), 7.36 (t, *J* = 7.31 Hz, 1H), 7.21 (dd, *J* = 5.38, 8.39 Hz, 2H), 7.02 (m, 4H), 6.01 (dd, *J* = 3.23, 11.40 Hz, 1H), 5.13 (s, 2H), 3.81 (dd, *J* = 11.40, 17.42 Hz, 1H), 3.16 (dd, *J* = 3.66, 17.42 Hz, 1H) ^{13}C NMR (176 MHz, CHLOROFORM-d) δ 176.2, 162.1(d, *J*=246.68, CH), 161.1, 155.7, 137.6(d, *J*=2.54 Hz, CH), 136.1, 128.8(CH), 128.7(CH), 128.2(CH), 127.4(CH), 127.3 (d, *J*=7.63 Hz, CH), 123.2, 115.7(d, *J*=22.89 Hz, CH), 115.2(CH), 77.2, 76.8, 70.1 (CH_2), 62.7 (CH), 43.1 (CH_2) IR (cm^{-1}) neat 3477, 3354 MS (APCI) m/z: 406.1 $[M+H]^+$ m.p. 199-200 ˚C HRMS calculated for $C_{23}H_{21}FN_3OS^+$: 406.1384 $[M+H]^+$ found 406.1384

3-(4-(benzyloxy)phenyl)-5-(2-chlorophenyl)-4,5-dihydro-1H-pyrazole-1-carbothioamide (SSE15213): 54% ^{1}H NMR (700 MHz, CHLOROFORM-*d*) δ 7.67 (d, *J* = 7.96 Hz, 2), 7.37 - 7.45 (m, 4H), 7.32 - 7.37 (m, 1H), 7.19 - 7.24 (m, 2H), 7.07 (s, 1H), 7.00 (d, *J* = 7.96 Hz, 2H), 6.33 (d, *J* = 11.19 Hz, 1H), 5.12 (s, 2H), 3.90 (dd, *J* = 11.62, 17.42 Hz, 1H), 3.10 (d, *J* = 17.64 Hz, 1H) ^{13}C NMR (176 MHz, CHLOROFORM-d) δ 176.3, 161.0, 156.1, 138.6, 136.1, 131.3, 130.0(CH), 128.8(CH), 128.7(CH), 128.6(CH), 128.2(CH), 127.4(CH), 127.2(CH), 123.1, 115.1(CH), 70.1(CH_2), 61.2(CH), 42.0 (CH_2) IR (cm^{-1}) neat 3414, 3257 MS (APCI) m/z: 422.1 $[M+H]^+$ m.p. 203-204 ˚C HRMS calculated for $C_{23}H_{21}ClN_3OS^+$: 422.1088 $[M+H]^+$ found 422.1088

3-(4-((4-methylbenzyl)oxy)phenyl)-5-phenyl-4,5-dihydro-1H-pyrazole-1-carbothioamide (SSE15214): 43% ^{1}H NMR (700 MHz, CHLOROFORM-*d*) δ 7.67 (d, *J* = 8.82 Hz, 2H), 7.35-7.32 (m, 4H), 7.25 - 7.28 (t, *J* = 7.31 Hz, 1H), 7.22 (dd, *J* = 7.53, 11.62 Hz, 4H), 7.00 (d, *J* = 8.82 Hz, 2H), 6.03 (dd, *J* = 3.66, 11.40 Hz, 1H), 5.08 (s, 2H), 3.81 (dd, *J* = 11.40, 17.42 Hz, 1H), 3.18 (dd, *J* = 3.55, 17.53 Hz, 1H), 2.37 (s, 3H) ^{13}C NMR (176 MHz, CHLOROFORM-d) δ 176.2, 161.1, 155.8, 141.8, 138.1, 133.1, 129.3(CH), 128.9(CH), 128.6(CH), 127.6(CH), 125.4(CH), 123.2, 115.1(CH), 70.0(CH_2), 63.3(CH), 43.1(CH_2), 21.2(CH_3) IR (cm^{-1}) neat 3391, 3257 MS (APCI) m/z: 402.2 $[M+H]^+$ m.p. 206-208 ˚C HRMS calculated for $C_{24}H_{24}N_3OS^+$: 402.1635 $[M+H]^+$ found 402.1635

5-(4-fluorophenyl)-3-(4-((4-methylbenzyl)oxy)phenyl)-4,5-dihydro-1H-pyrazole-1-carbothioamide (SSE15215): 61% ^{1}H NMR (700 MHz, CHLOROFORM-*d*) δ 7.74 (d, *J* = 9.03 Hz, 2H), 7.40 (d, *J* = 7.74 Hz, 2H), 7.26 - 7.30 (m, 4H), 7.10-7.08 (m, 4H), 6.08 (dd, *J* = 3.44, 11.19 Hz, 1H), 5.16 (s, 2H), 3.88 (dd, *J* = 11.40, 17.42 Hz, 1H), 3.23 (dd, *J* = 3.66, 17.42 Hz, 1H), 2.45 (s, 3H) ^{13}C NMR (176 MHz, CHLOROFORM-*d*) δ 176.2, 162.0(d, *J* = 246.68 Hz, CH), 161.1, 155.7, 138.1, 137.6(d, *J* = 3.81 Hz, CH), 133.1, 129.3 (CH), 128.6, (CH) 127.6 (CH), 127.2(d, *J* = 7.63 Hz, CH) , 123.0, 115.7(d, *J* = 21.62 Hz, CH), 115.2 (CH), 70.0(CH_2), 62.7 (CH), 43.1(CH_2), 21.2(CH_3) IR (cm^{-1}) neat 3476, 3353 MS (APCI) m/z: 420.2 $[M+H]^+$ m.p. 192-194 ˚C HRMS calculated for $C_{24}H_{23}FN_3OS^+$: 420.1540 $[M+H]^+$ found 420.1540

5-(4-chlorophenyl)-3-(4-((4-methylbenzyl)oxy)phenyl)-4,5-dihydro-1H-pyrazole-1-carbothioamide (SSE15216): 52% ^{1}H NMR (700 MHz, CHLOROFORM-*d*) δ ppm 7.66 (d, *J*=8.39 Hz, 2H) 7.33 (d, *J*=7.53 Hz, 2H) 7.30 (d, *J*=7.96 Hz, 2H) 7.22 (d, *J*=7.53 Hz, 2H) 7.17 (d, *J*=8.17 Hz, 2H) 7.01 (d, *J*=8.60 Hz, 2H) 5.99 (dd, *J*=11.29, 2.90 Hz, 1H) 5.08 (s, 2H) 3.80 (dd, *J*=17.53, 11.51 Hz, 1H) 3.13 (dd, *J*=17.53, 3.12 Hz, 1H) 2.38 (s, 3H) ^{13}C NMR (176 MHz, CHLOROFORM-*d*) δ ppm 176.2, 161.1, 155.6, 140.3, 138.0, 133.2, 133.1, 129.3 (CH), 129.0 (CH), 128.6 (CH), 127.5 (CH), 126.9 (CH), 123.0, 115.2 (CH), 70.0 (CH_2), 62.7 (CH), 42.9 (CH_2), 21.2 (CH_3) IR (cm^{-1}) neat 3482, 3359 MS (APCI) m/z: 436.1 $[M+H]^+$ m.p. 146-147 ˚C HRMS calculated for $C_{24}H_{23}ClN_3OS^+$: 436.1245 $[M+H]^+$ found 436.1245

Full length Blots:

All the blots used in this paper were either from different gels or different parts of the same gels (For proteins with different molecular weights). Each blot with an indicated antibody has been delineated with a black border. Nowhere in this manuscript two different parts of the same blot (either from the same gel or from different gels) have been put together. For proteins with different molecular weights, gels were cut before blotting and the full blots are presented below.

Figure 2A

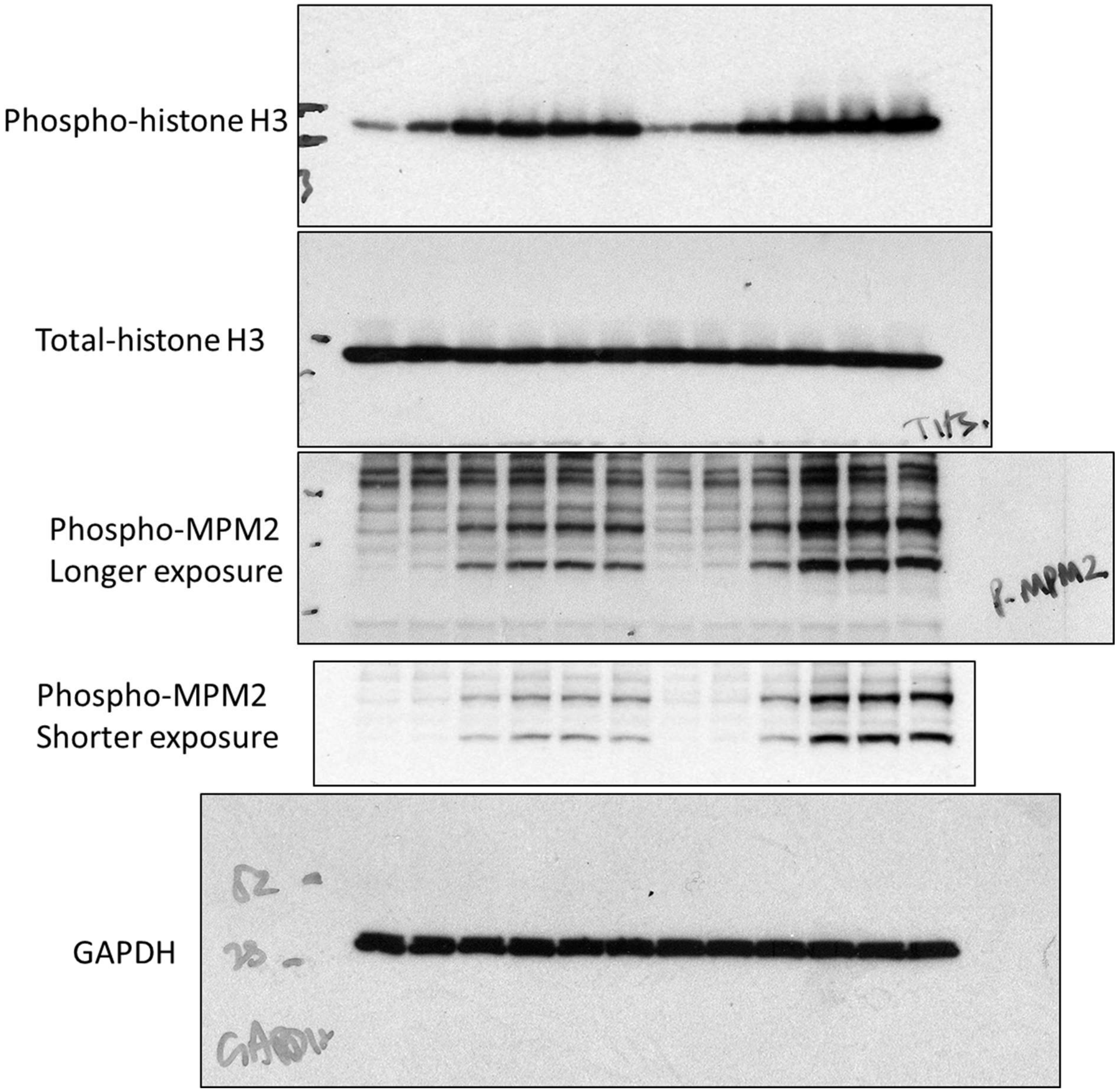
Phospho-histone H3
Total-histone H3
Phospho-MPM2
Longer exposure
Phospho-MPM2
Shorter exposure
GAPDH

Figure 4A

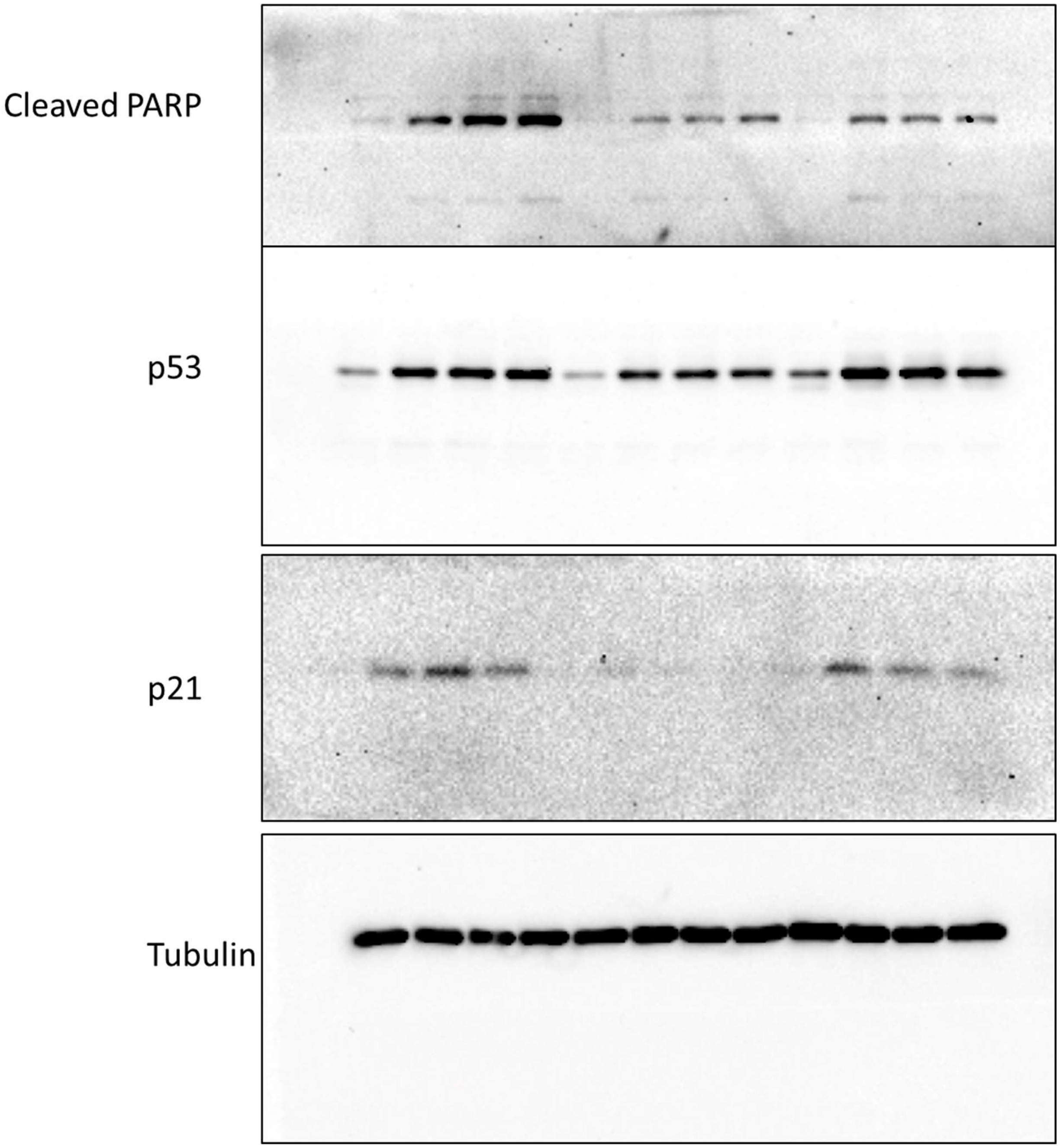

Figure 5A

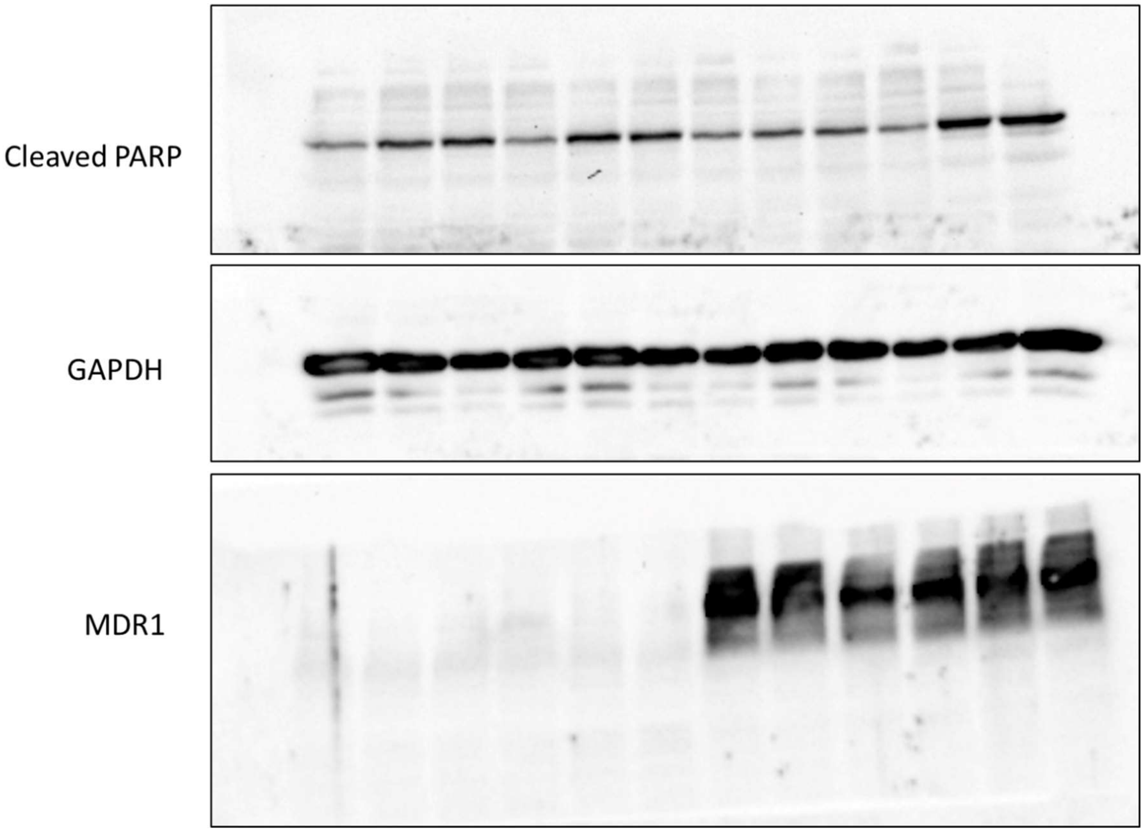

Figure 5A

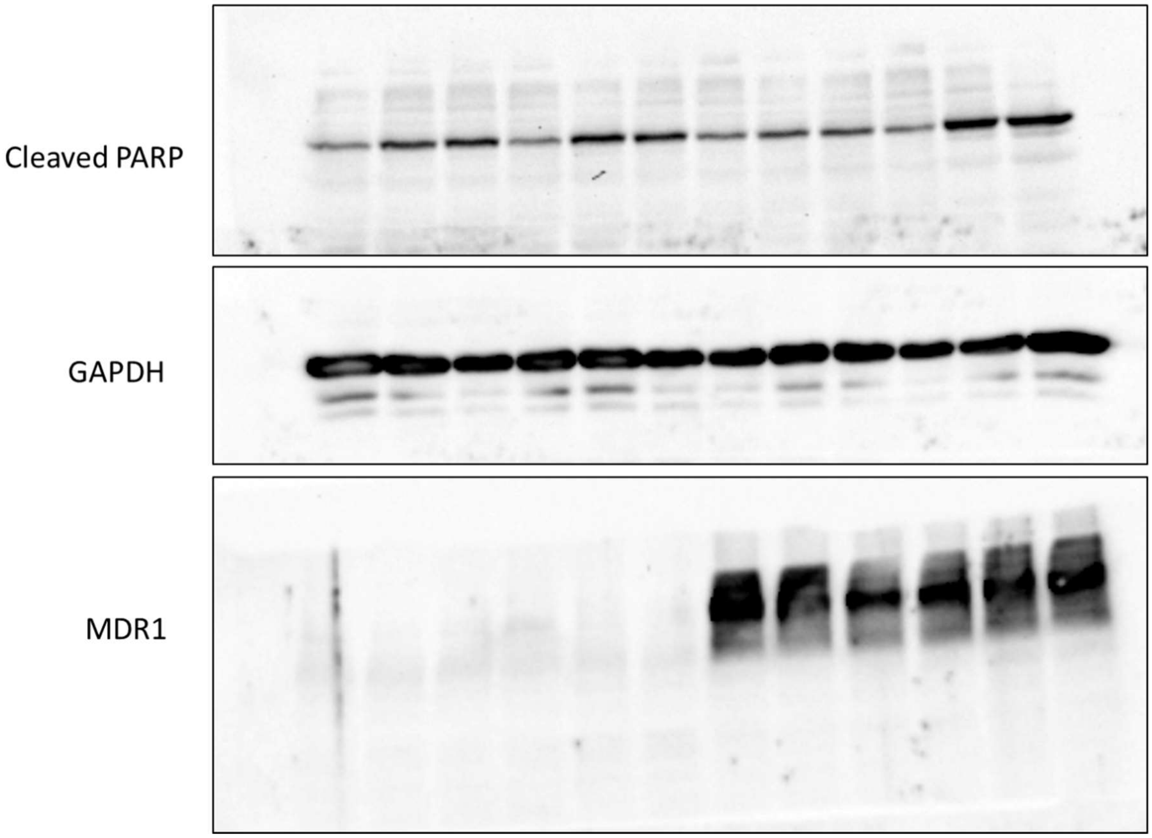

Figure 5B

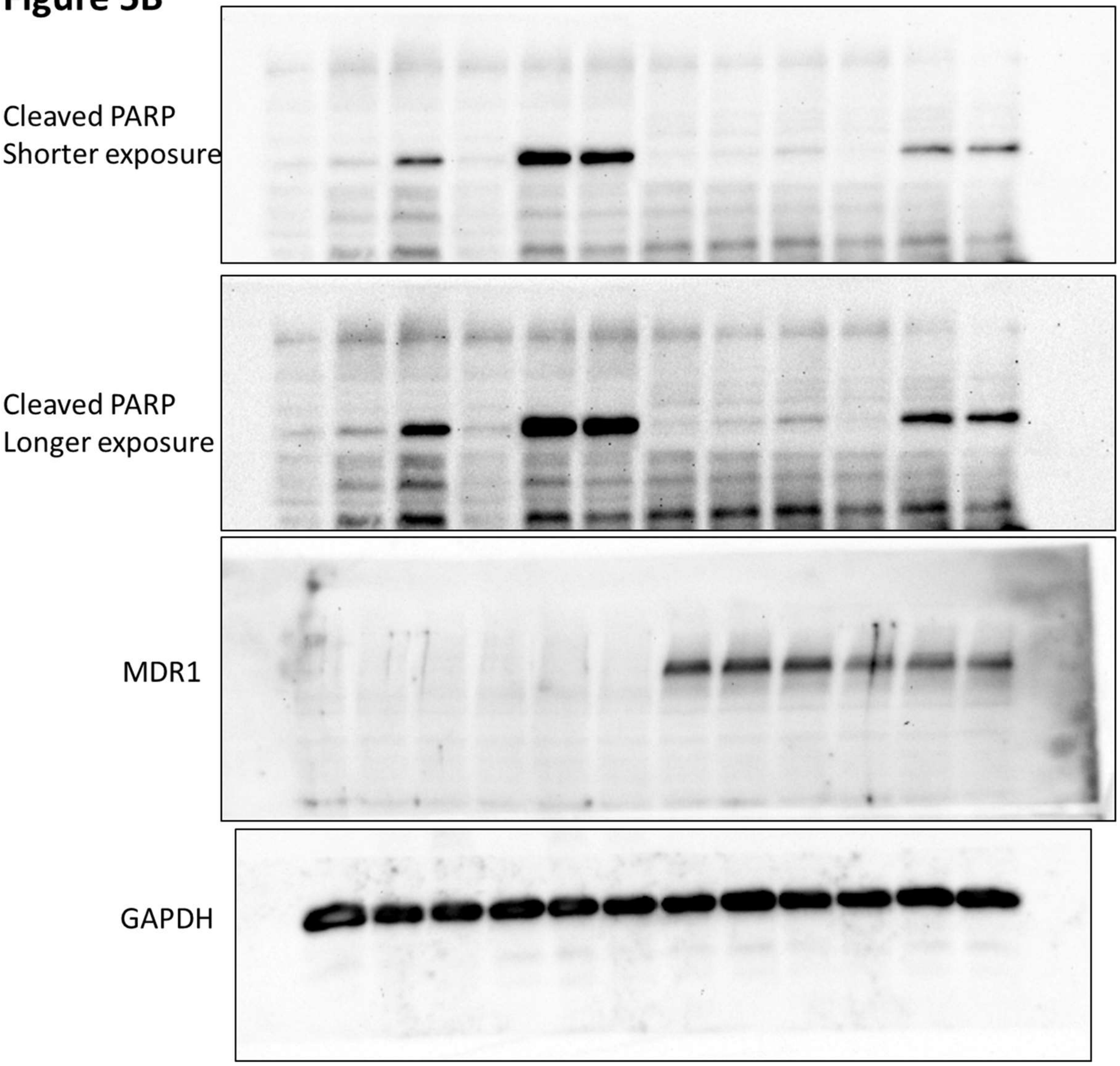
Cleaved PARP
Shorter exposure
Cleaved PARP
Longer exposure
MDR1
GAPDH

Supplementary Figure 1

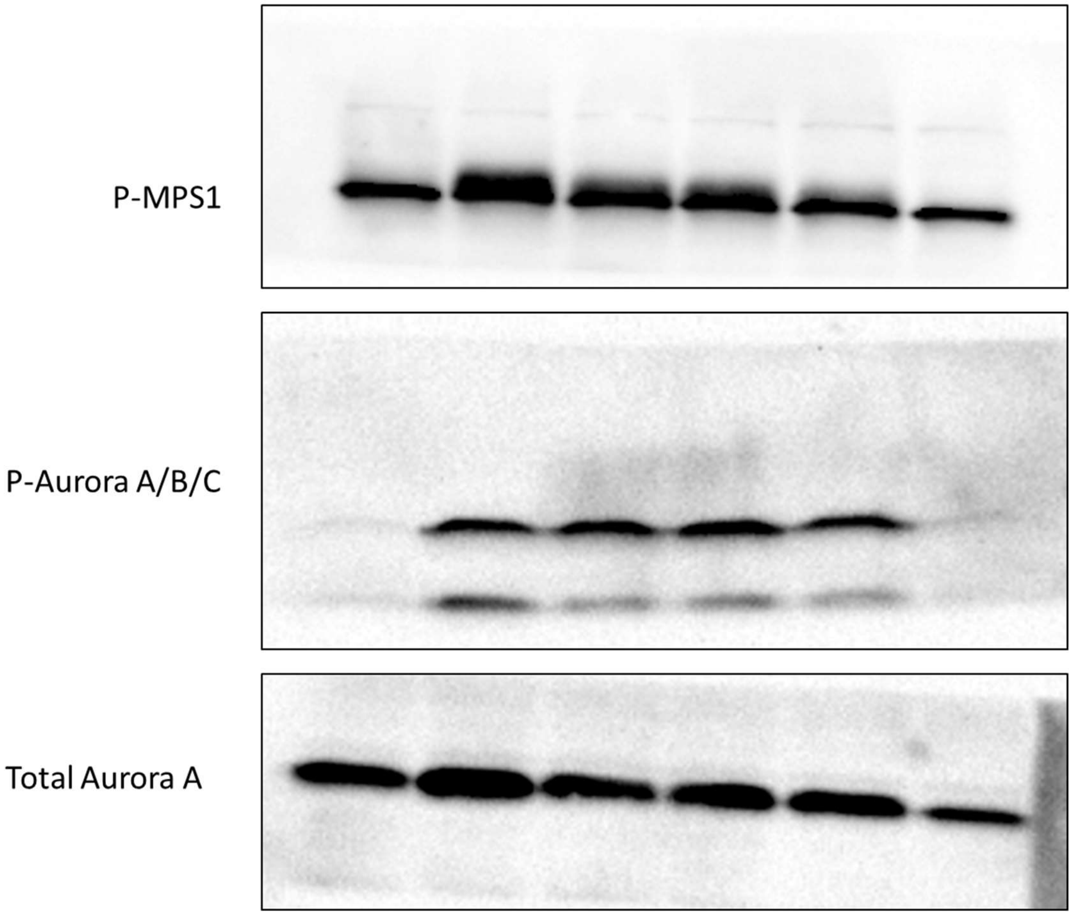

1 Zhang, Y. L. *et al.* Synthesis and Biological Evaluation of 1-Methyl-1H-indole–Pyrazoline Hybrids as Potential Tubulin Polymerization Inhibitors. *ChemMedChem* **11**, 1446-1458 (2016).

2 Abdel-Aziz, M., Aly, O. M., Khan, S. S., Mukherjee, K. & Bane, S. Synthesis, Cytotoxic Properties and Tubulin Polymerization Inhibitory Activity of Novel 2-Pyrazoline Derivatives. *Archiv der Pharmazie* **345**, 535-548 (2012).
